# Microalgal polyunsaturated fatty acids: Hotspots and production techniques

**DOI:** 10.3389/fbioe.2023.1146881

**Published:** 2023-03-31

**Authors:** Weixian Chen, Tianpei Li, Shuwen Du, Hui Chen, Qiang Wang

**Affiliations:** ^1^ State Key Laboratory of Crop Stress Adaptation and Improvement, School of Life Sciences, Henan University, Kaifeng, China; ^2^ School of Food Science and Engineering, Wuhan Polytechnic University, Wuhan, China; ^3^ Academy for Advanced Interdisciplinary Studies, Henan University, Kaifeng, China

**Keywords:** algae, PUFA, research hotspots, extraction, enrichment, omega-fatty acids

## Abstract

Algae play a crucial role in the earth’s primary productivity by producing not only oxygen but also a variety of high-value nutrients. One such nutrient is polyunsaturated fatty acids (PUFAs), which are accumulated in many algae and can be consumed by animals through the food chain and eventually by humans. Omega-3 and omega-6 PUFAs are essential nutrients for human and animal health. However, compared with plants and aquatic sourced PUFA, the production of PUFA-rich oil from microalgae is still in the early stages of exploration. This study has collected recent reports on algae-based PUFA production and analyzed related research hotspots and directions, including algae cultivation, lipids extraction, lipids purification, and PUFA enrichment processes. The entire technological process for the extraction, purification and enrichment of PUFA oils from algae is systemically summarized in this review, providing important guidance and technical reference for scientific research and industrialization of algae-based PUFA production.

## Introduction

Algae are a group of photosynthetic organisms that evolved earlier than higher plants. Organisms that contain chlorophyll a in cells but lack differentiation of roots, stems, leaves and other tissues and organs are generally classified as algae (Lee, 2018). Humans have been utilizing algae as food for thousands of years, such as *Nostoc*, *Aphanizomenon* and *Spirulina* (Jensen et al., 2001). Macroalgae are typically consumed directly by humans, while microalgae are indirectly consumed through the consumption of fish and other animals. Algae are the primary contributors to water productivity on Earth, providing nutrients for many aquatic organisms (Chen H et al., 2021). Some algae are particularly suitable as feed (Becker, 2003a) for animals, and initial processing techniques have been established (Amorim et al., 2020).

Microalgae are a valuable source of biologically active substances and a treasure trove of resources. Recent articles have highlighted their ability to produce a variety of valuable products such as lipids, proteins, sugars, mannan (Capek et al., 2023), chitin (Chiriboga and Rorrer, 2019), terpenoids (Huang et al., 2021), vitamins (Ljubic et al., 2021). Some microalgae also produce essential fatty acids like alpha-linolenic acid (ALA) (Meng et al., 2018), eicosapentaenoic acid (EPA) (Peltomaa et al., 2018; Xia et al., 2020), docosahexaenoic acid (DHA) (Peltomaa et al., 2018), gamma-linolenic acid (GLA) (Ronda et al., 2012).

The analysis shows that lipids and fatty acids are the prominent research areas in algae research. Algal cells typically contain lipids ranging from 1% to 40% of their dry weight, while some species can accumulate up to 85% (Becker, 2003b; Chen and Wang, 2021). These lipids generally exist in cells in the form of glycerides, free fatty acids, glycolipids and phospholipids (Becker, 2003b). Certain algae species can produce both PUFA and pigment fucoxanthin simultaneously, such as diatom *Nitzschia laevis* (Lu et al., 2019), *Tisochrysis lutea* (Gao et al., 2022). Apart from nutrition and health applications, algae also have significant potential in environmental fields, such as biodiesel production (Zhu et al., 2016; Chen and Wang, 2022), hydrogen production (Chen et al., 2023), as well as the removal of nitrogen (Chen et al., 2016; Wang J et al., 2019; Chen and Wang, 2020), flame retardants (Zhang et al., 2021), and organics (Zheng et al., 2021) from wastewater. However, compared with fossil energy, due to the high cost of production, extraction, and conversion, algae biodiesel’s commercial advantages are limited (Chen et al., 2019; Chen H et al., 2020). Therefore, since 2014, the focus of algae research has gradually shifted towards high-value bioproducts and environmental applications (Rashid et al., 2018).

**FIGURE 1:**
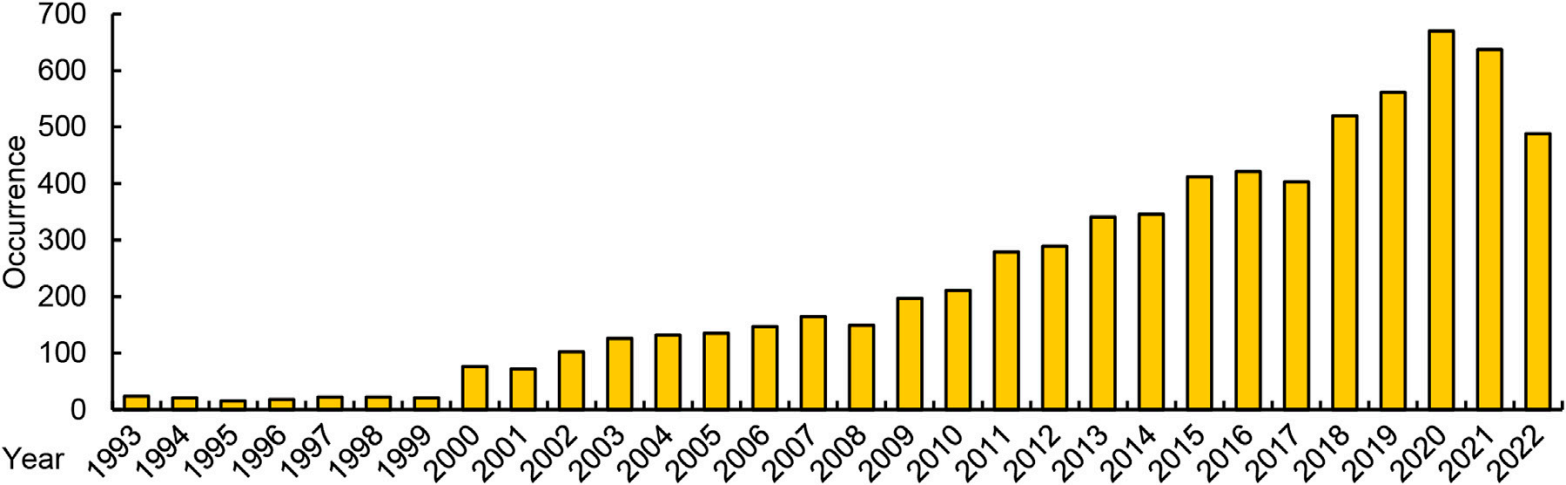
The number of *algae*-*PUFA*-related research reports in recent years in the web of science.

Algae are currently being developed as new sources of the polyunsaturated fatty acids (PUFAs), including linolenic acid (LA), ARA, EPA, docosapentaenoic acid (DPA), DHA (Chen W et al., 2021; Cui et al., 2021). Algal DHA has already been commercialized in the market. Although the deep-sea fish oil PUFAs currently available are derived from fish, they are essentially derived from the oil accumulated by algae ingested by fish (Ebm et al., 2021). To identify related research, we collected keywords related to *algae* and *PUFA*, and the 41 kinds of unsaturated fatty acids (UFAs) introduced on Wikipedia. Using these keywords, we searched the Web of Science and retrieved over 7,000 reports related to algae-PUFA research. From the perspective of statistics in Figure 1, *algae-PUFA* research is an important research hotspot and has shown continuous growth in recent years.

## Analysis of research hotspots

**FIGURE 2:**
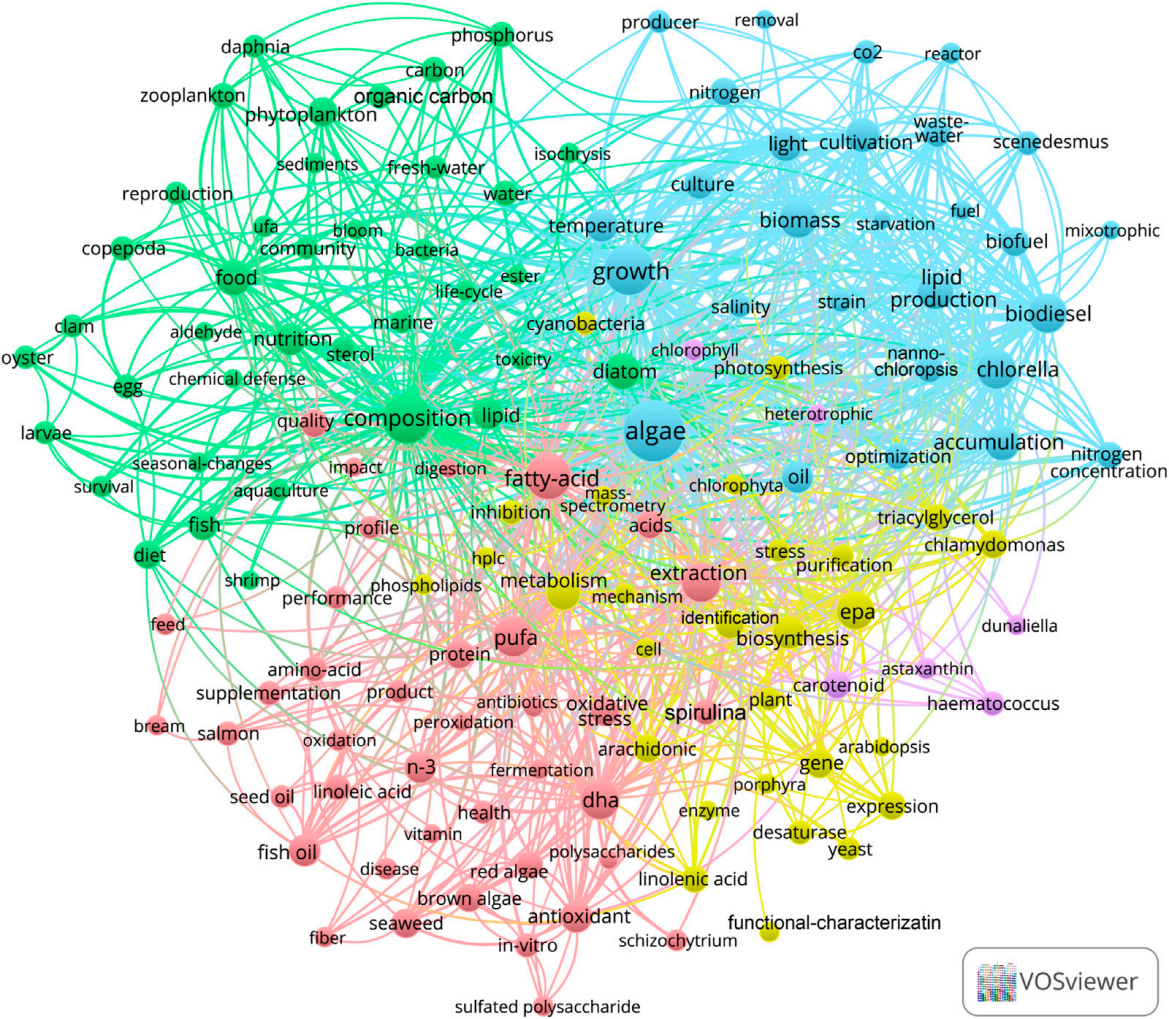
The most popular keywords in the articles of *algae-PUFA* in the Web of Science Core Collection.

The VOS viewer (version 1.6.18) was used to analyze the keywords found in the retrieved reports. Among the keywords, *algae* was found to be the central theme, and the keywords most closely related to *algae* were *fatty*-*acid*, *composition*, *growth*, and *extraction* (as shown in Figure 2).

In the blue cluster depicted in Figure 2, *growth* is found to be most closely related to *light*, *biomass*, *cultivation*, *lipid production*, *biodiesel*, *accumulation*. This indicates that there are some key factors involved in the accumulation of PUFA in the algae culture. Furthermore, algae oil has emerged as a critical area of research in the field of *biofuel* and *biodiesel*. However, the production of biodiesel from algae is considered to be expensive and less commercially viable (Kumar et al., 2013). With recent technological advancement, the production cost of microalgae biodiesel based on waste water and seawater has been reduced to US$2.2/kg, which is still much higher than that of fossil energy (Yu et al., 2023). Even after considering the revenues from wastewater treatment and carbon dioxide capture, the commercial competitiveness of microalgae biodiesel remains insufficient when compared with fossil energy. On the other hand, the production of PUFAs has proved to be commercially successful.

**FIGURE 3:**
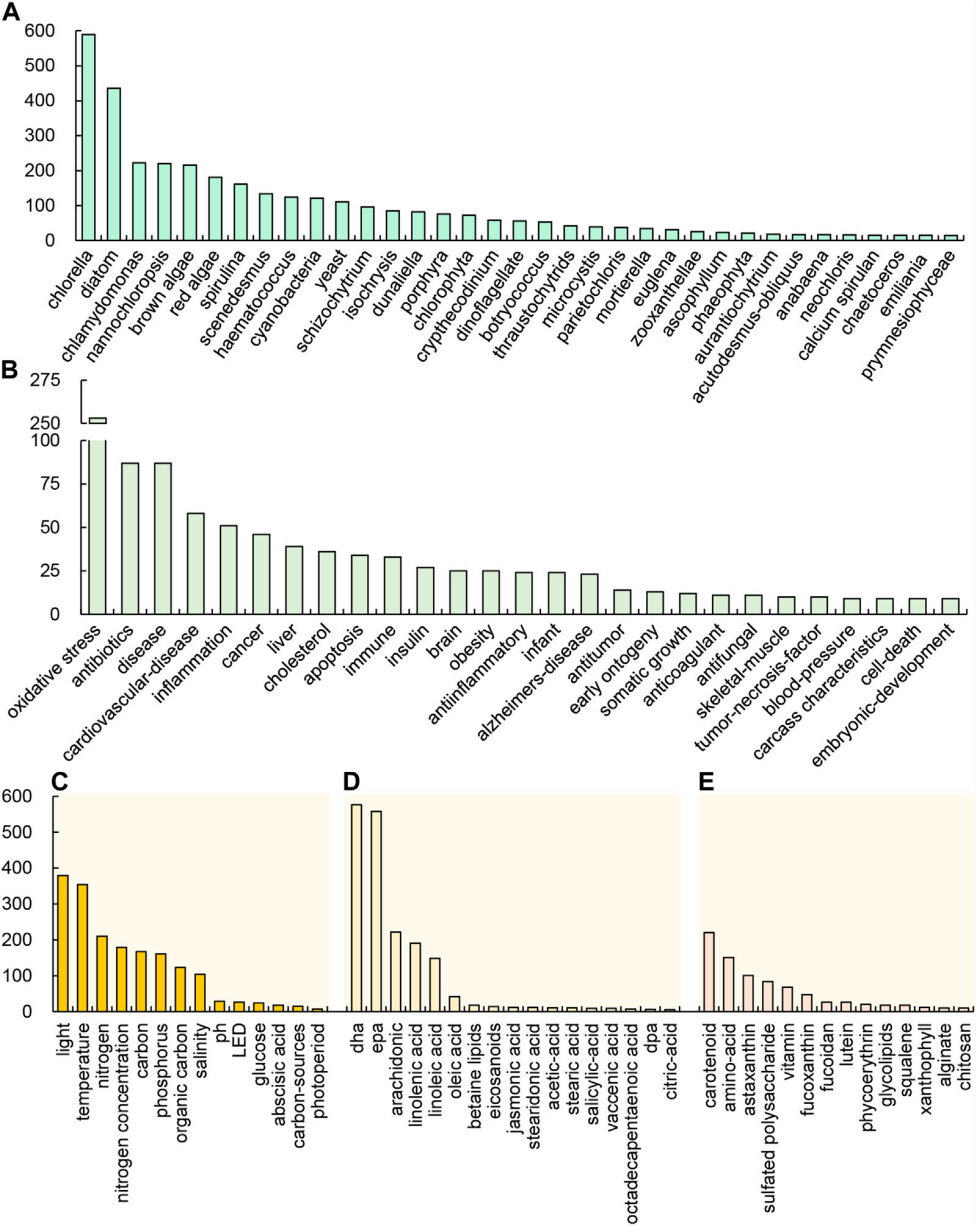
Occurrence of the main keywords in the reports of *algae-PUFA* according to the species **(A)**, health functions **(B)**, conditions **(C)**, kinds of PUFAs **(D)**, other high value products **(E)**.

The microorganisms associated with PUFAs were ranked based on their occurrence, with green algae and diatoms being relatively more frequent (Figure 3A). Among green algae, *Chlorella*, *Chlamydomonas*, *Nannochloropsis*, *Scenedesmus*, *Haematococcus*, *Dunaliella* were reported more quantitatively. Among diatoms, there were more reports of *Phaeodactylum tricornutum*, *Skeletononema costatum*, and *Thalassiosira rotula*. It suggests that research on PUFAs production is mostly concentrated in these algae. In general, diatoms can accumulate a large amount of long-chain PUFA in cells, but their biomass production rate is generally relatively low even when supplemented with organic carbon sources (Marella et al., 2021; Cheah et al., 2022). On the other hand, *Cryptheccdinium cohnii* and *Schizochytrium* have a high content of long-chain PUFAs such as DHA and EPA, as well as high biomass production rates. Some green algae, such as *Chromochloris zoffingiensis*, can accumulate astaxanthin, but the accumulated PUFAs is mainly C18 PUFAs (Liu et al., 2016; Mao et al., 2018). Therefore, some research groups are also attempting to co-produce long-chain PUFAs and astaxanthin in algae cells, making it more cost-effective for commercial production (Pawar et al., 2021).

The keywords related to the cultivation conditions were ranked based on their occurrence, as shown in Figure 3C. Among these keywords, *nitrogen* (*concentration*), *light*, *temperature*, *carbon*, *phosphorus*, *organic carbon*, *salinity*, *pH*, *LED*, and *glucose* were found to be the key research factors. It is evident that the nitrogen source is the most relevant or critical for algal PUFA production. Other environmental or nutritional factors may also affect the accumulation of PUFAs. Therefore, to optimize culture conditions and increase the yield of algae PUFAs, the above environmental factors can be considered for trial testing.

In the green cluster *composition* shown in Figure 2, the focus is mainly on some keywords that PUFAs affect animal nutrition. This also indicates that PUFAs plays an important role in improving the nutritional value of aquatic products. Furthermore, the accumulation of PUFAs in many aquatic products is also due to the PUFAs accumulated by algae in the water (Brett and MÜller-Navarra, 1997; Nichols, 2003).

In the pink cluster in Figure 2, the keywords with higher occurrence are *fatty-acid*, *PUFA*, and *extraction*. These keywords are mainly related to the health benefits of PUFAs. *Extraction* is an important aspect of *algae-PUFA* production research, and closely related to *growth*, *composition*, *PUFA*, *carotenoid*. Additionally, in the exploration of algae biodiesel production, extraction is a significant cost factor (Kumar et al., 2013; Rashid et al., 2018).

In the yellow cluster in Figure 2, the keywords with higher occurrence are *EPA* and *biosynthesis*, which are related to the synthesis of PUFAs. The keywords of interest include *desaturase* and *linolenic acid*. Linolenic acid is not only an important PUFA, but also a precursor for the synthesis of other PUFAs, such as DHA and EPA. The human body has the ability to convert linolenic acid into other PUFAs. Ensuring the intake of linolenic acid may be beneficial for human health and meet the body’s demand for longer chain PUFAs such as DHA and EPA. In addition, the occurrence of various PUFA types is ranked by occurrence in Figure 3D, which shows that DHA and EPA have the highest occurrence. The occurrences of arachidonic acid (ARA), linolenic acid, and linoleic acid are also relatively high. The occurrence of oleic acid is very small, and the occurrence of other PUFAs is very low. This implies that the application value of these PUFAs is related to the current market demand.

In the purple cluster in Figure 2, *carotenoid* was the important keyword, closely related to *astaxanthin*, *Haematococcus* and *Dunaliella*, followed by *heterotrophic* and *chlorophyll*. Moreover, both carotenoid and PUFAs in the pink cluster are closely related to *antioxidant*. The keywords of other high-value products are ranked by their occurrences (Figure 3E). It can be seen that *carotenoid*, *amino*-*acid*, and *astaxanthin* are other high-value products with the highest correlation with *algae-PUFA*. This may be because the accumulation of these substances has a certain correlation with the accumulation of PUFAs. The simultaneous accumulation of PUFAs with other high-value products can greatly increase the potential for practical production applications of these algae.

## Sources of PUFAs

### Synthesis

Fatty acids are long-chain carboxylic acids that serve as crucial raw materials for oils and directly affect the properties and functions of oils. Fatty acids can be divided into saturated fatty acids and unsaturated fatty acids (UFAs). Fatty acids that lack carbon-carbon double bonds in the aliphatic chain are referred to as saturated fatty acids, which include palmitic acid and stearic acid, among others. PUFAs are straight-chain fatty acids with two or more double bonds (Metzner, 1984). Depending on the position of the first double bond from the methyl end of the fatty acid, PUFAs can be classified into ω-x fatty acids, including ω-9, ω-6, ω-3 (also referred to as n-9, n-6, n-3) (Djuricic and Calder, 2021).

**FIGURE 4:**
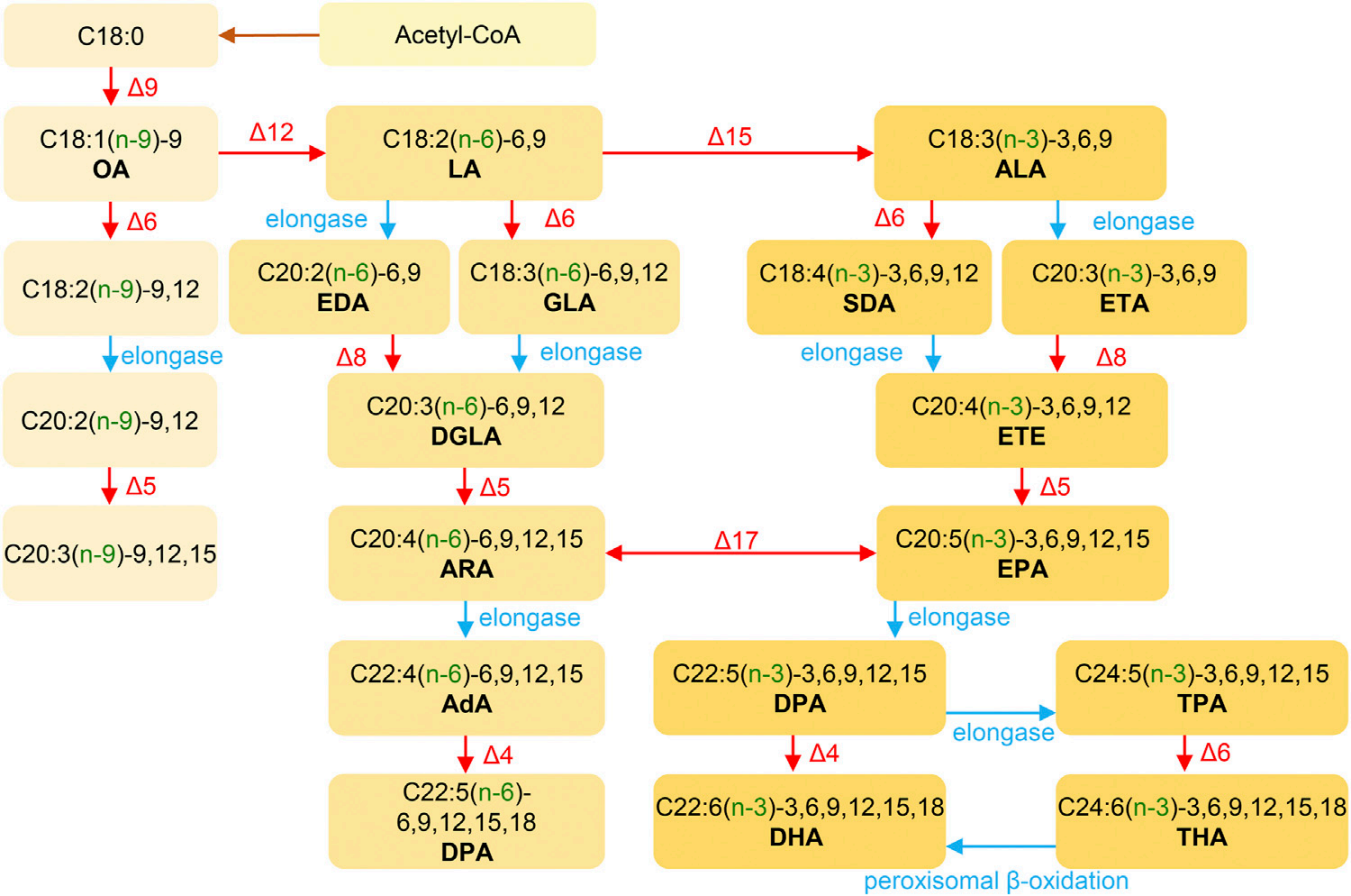
The general pathways of PUFAs synthesis. Desaturase: Δ4/Δ5/Δ6/Δ9/Δ15/Δ17; C18/20/22, carbon numbers; :1/:2/…/:6, number of olefinic bonds; (n-3/6/9), double bond between the (n–x) and (n-x+1) to last carbon atoms; −3/6/9/12/15/18, double bond between the x and x+1 to last carbon atoms. AdA, adrenic acid; ALA, α-linolenic acid; ARA, arachidonic acid; DGLA, dihomo-γ-linolenic acid; DHA, docosahexaenoic acid; DPA, osbond acid; DPA, Docosapentaenoic acid; EDA, eicosadienoic acid; EPA, eicosapentaenoic acid; ETA, eicosatrienoic acid; ETE, eicosatetraenoic acid; GLA, γ-linolenic acid; LA, linoleic acid; OA, oleic acid; SDA, stearidonic acid; THA, tetracosahexanoic acid; TPA, tetracosapentanoic acid.

The biosynthesis of ω-3, ω-6 and ω-9 PUFAs begins with acetyl-CoA and requires a series of carbon chain elongation and desaturation reactions, as illustrated in Figure 4 (Uemura, 2012; Scanferlato et al., 2019). The synthesis of ω-3 and ω-6 polyunsaturated fatty acids follows two distinct pathways, and intermediates from one pathway cannot be directly converted into intermediates of the other (Napier, 2002). The ω-3 PUFAs like DHA and EPA are derived from ALA, while the ω-6 PUFAs, such as GLA and ARA are derived from LA (9,12-linoleic acid) (Lehninger, 1982). The synthesis of PUFAs in microorganisms is similar to that in higher organisms. Acetyl-CoA and malonyl-CoA form LA under the action of fatty acid synthase complex, which is then further metabolized to PUFAs through the ω-6 and ω-3 pathways (Lehninger, 1982).

Humans and other mammals are incapable of synthesizing ω-3 and ω-6 PUFAs *de novo* due to the absence of Δ12 and Δ15 dehydrogenases (Napier, 2002). Therefore, LA and ALA must be obtained from dietary sources (Djuricic and Calder, 2021). The synthetic pathways of ω-3 and ω-6 PUFAs share common desaturases and chain-elongation enzymes, resulting in a metabolic competition process (Smink et al., 2012). Therefore, a balanced intake of LA and ALA is crucial. The essential fatty acid ALA serves as a precursor to ω-3 PUFAs such as EPA and DHA (Brenna et al., 2009). Thus, supplementing these precursors can help to increase EPA or DHA levels, with a more comprehensive supplementing effect.

### Main physiological and health functions

The health benefits of omega-3 PUFA was initially studied through the correlation between fish oil intake and health risks, mainly related to reducing triglycerides, lowering blood pressure, preventing thrombosis, and interfering with arachidonic acid cascade reaction, and thus playing an anti-atherosclerosis role (Simopoulos, 1991; Kris-Etherton et al., 2002). EPA and DHA have shown promising results in prevention, body weight management, and cognitive function in patients with mild Alzheimer’s disease (Swanson et al., 2012). Figure 3B displays the occurrence of keywords related to health and disease, where the *antioxidant* activity of PUFAs is the most prominent characteristic. PUFAs has anti-oxidation and anti-aging effects. Clinical studies have shown that after taking ALA, the activity of glutathione peroxidase and superoxide dismutase increases, the generation of malondialdehyde decreases, and it has an antioxidant effect (Mahsa et al., 2017; Boskabady et al., 2019; Korbecki et al., 2019). In addition, PUFAs have reported functions in antibiotics, cardiovascular-disease, anti-inflammation, cancer, liver health, blood lipids, diabetes, brain health, and ontogeny. In summary, PUFAs play an important role in human health, with their main physiological functions related to the circulatory system, immune system and nervous system. A summary is shown in Table 1. Omega-3 PUFAs have a strong market presence due to their crucial structural and physiological functions, as well as their beneficial health effects.

**TABLE 1:** The main function of PUFAs.

Main functions	Physiological function
**Circulatory System**	
Regulate Blood Lipids	Lower blood pressure (Paschos et al., 2007; Hashimoto et al., 2020) and have protective effect on the heart (Rajaram, 2014).
Antithrombotic	Reduce the risk of some coronary heart disease and cerebral ischemia (Winnik et al., 2011; Stivala et al., 2013; Rajaram, 2014), reduce the risk of atherosclerosis (Varin et al., 2015), and effectively prevent and treat thrombotic diseases (Yamashita et al., 2005; Golanski et al., 2021).
Effects On Insulin Resistance	ALA intake was inversely associated with insulin resistance, whereas DHA combined with EPA was not (Muramatsu et al., 2010). ALA helps control blood sugar in type II diabetic rats (Rajaram, 2014; Mejía-Zepeda and Pérez-Hernández, 2020).
**Immune System**	
Anti-Inflammatory, Anti-Autoimmune & Anti-Allergic	Treating and preventing allergic dermatitis, bronchial asthma and pollen allergy (James et al., 2000; Calder, 2002). Inhibiting the body’s allergic reaction (Boskabady et al., 2019; Kaveh et al., 2019), restoring and improving immunity are important treatments against inflammation (Winnik et al., 2011; Kaveh et al., 2019). Positive effect in the treatment of alcoholic liver disease and inflammation of the liver (Wang M et al., 2019).
Antitumor	Inhibits the growth and metastasis of breast cancer (Corsetto et al., 2011; Yang et al., 2014).
**Nervous System**	
Central Nervous System	Constitute brain nerve cells, retinal and other tissue cells (Abedin et al., 1999).
	Promote the synthesis of proteins and certain neurotransmitter substances in the brain, and play an important role in the growth of brain nerve cells and neurotransmitter synapses (Jeffrey et al., 2002a; Jeffrey et al., 2002b).
	Promote the development of the nervous and visual system of infants and young children (Guesnet and Alessandri, 2011; Muhlhausler et al., 2018), and promote the formation of vision (Youdim et al., 2000; Mizukami et al., 2018).
	It has an effect on maintaining and improving learning and memory ability (Umezawa et al., 1999; Petursdottir et al., 2008; de Souza et al., 2011).
Visual System	An important structural lipid component of retinal tissue (Salem Jr et al., 1986), which helps maintain good vision (Neuringer et al., 1986). Lack of DHA, the vision is greatly reduced in the late growth period (Bell et al., 1995).
Alzheimer’s Disease	For Alzheimer’s dementia, PUFAs can normalize brain tissue in the state of DHA deficiency, and the anti-inflammatory effect of DHA can improve the immune response in the brain (Swanson et al., 2012; Devassy et al., 2016; Fonteh et al., 2020).

### Food sources of PUFAs

According to Figure 4, ALA is the primary precursor of ω-3 PUFAs, and is mainly used in the pharmaceutical, food and cosmetic industries, with potential for even more new uses. Currently, the largest use of ALA is in dietary nutrition. The Center for Genetics, Nutrition and Health of the National Institutes of Health in the United States recommends a daily intake is 2.22 g/day of ALA (Simopoulos et al., 2000). However, ALA is found in low amounts in common foods, including various fresh vegetables. The content of ALA in plants is generally around 100 mg/100 g (fresh weight), and is mainly present in glycolipids in the leaves (Raper et al., 1992; Ayerza, 1995). Common sources and contents of ALA are shown in Table 2.

**TABLE 2:** Contents (%) of UFAs in total lipid of common foods.

Sources	OA	LA	GLA	ALA	EPA	DHA	Reference
Flaxseed oil	∼21	∼13	<1	43–61	<1	<1	Aguillón-Páez et al. (2020), Yang *et al.* (2021)
Refined *Canola* seed oil	∼61	∼25	<1	∼7	<1	<1	Özcan *et al.* (2021)
Refined linoleic sunflower oil	∼34	∼54	—	—	—	—	Aşkın and Kaya (2020)
Oyster nut oil	∼9	∼49	<1	<1	<1	<1	Mwakasege *et al.* (2021)
Extra-virgin olive oil	55–83	3–21	—	<1	<1	<1	Gunstone (2011)
Soybean oil	∼22	∼56	—	∼7	—	—	Rodríguez-Miranda *et al.* (2019)
Walnuts Oil	∼8	∼61	—	∼12	—	—	Copolovici *et al.* (2017)
Butter lipids	15–18	1–2	<1	<1	—	—	Faraji Sarabmirza *et al.* (2023)
Milk lipids	39–45	9–13	<1	<1	—	—	Anzhany *et al.* (2021)
Beef lipids	40–42	2–3	<1	<1	<1	<1	Najar-Villarreal *et al.* (2019)
Muscle lipids of farmed Salmon	∼34	∼10	∼12	∼3	∼10	—	Hatlen *et al.* (2022)
Spinach leaves	—	—	—	∼0.14 (DW)	—	—	Rajaram (2014)

DW, dry weight.

ALA-rich products available in the market are typically sourced from a few crops, such as flaxseed, as well as deep-sea fish oil. Marine fish are unable to synthesize DHA on their own. Instead, the DHA contained in their bodies originates from marine microorganisms, such as phytoplankton, and accumulates in marine fish, reptiles and mollusks through the marine food chain (Brett and MÜller-Navarra, 1997; Nichols, 2003). Research has shown that certain marine bacteria, algae, and fungi are capable of synthesizing PUFAs (Ratledge, 2001). Some algae, in particular, can accumulate ALA ranging from 10% to 40% of the total lipid fraction, as shown in Table 3.

**TABLE 3:** The content (%) of PUFAs in total lipid of some algae strains.

Organisms	GLA	ALA	EPA	DHA	Reference
*Acutodesmus obliquus*	—	∼38	—	—	Othman *et al.* (2019)
*Aurantiochytrium* sp.	—	—	∼1	∼42	Chauhan *et al.* (2023)
*Chlamydomonas reinhardtii*	—	∼44	—	—	Zheng *et al.* (2022)
*Chlorella sorokiniana*	—	∼11	∼2.4	—	Shim *et al.* (2020)
*Chlorella vulgaris*	—	∼35.4	—	—	Othman et al. (2019)
*Chlorococcum (Oophila) amblystomatis*	∼4	∼31	—	—	Correia *et al.* (2020)
*Codium fragile* (Suhr) Hariot (Chlorophyta)	—	14.2–19.9	3.0–4.4	—	Schmid *et al.* (2014)
*Codium tomentosum*	∼3	∼12	∼2	∼12	Ferreira *et al.* (2022)
*Crypthecodinium cohnii*	—	—	—	37–40	Moniz *et al.* (2021)
*Isochrysis galbana*	—	∼6	∼3	∼10	Señoráns *et al.* (2020)
*Isochrysis zhangjiangensis*	—	∼11	∼2	∼14	Li Y et al. (2019)
*Isochrysis galbana*	—	∼11	∼7	∼14	Ge *et al.* (2023)
*Nannochloropsis* sp.	—	0.1–17	4–34	—	Ma *et al.* (2016)
*Nannochloropsis oceanica*	—	—	∼27	—	Matsui *et al.* (2022)
*Oocystis pusilla*	—	23	—	—	Vidyashankar *et al.* (2015)
*Phaeodactylum tricornutum*	—	—	17.7	2.38	Wang X et al. (2019)
*Phaeodactylum tricornutum*	∼0.8	—	27–30	∼0.7	Kadalag *et al.* (2022)
*Scenedesmus* sp. HSJ296		∼42			Meng et al. (2018)
*Schizochytrium limacinum*	—	∼1	∼1	∼45	Rodríguez-España et al. (2022)
*Arthrospira platensis*	34.5	—	—	—	Yang X et al. (2019)
*Spirulina platensis*	15.8	—	—	—	Yang X et al. (2019)
*Thraustochytriidae* sp.	—	—	<8	∼21	Quilodrán *et al.* (2010)

Algae are the original source of ω-3 PUFAs in fish oil, making algae oil a crucial source of PUFAs. An increasing number of algae are now being cultivated on a large scale for the production of some bioactive substances, including PUFAs. Compared to planting terrestrial crops to produce oil to obtain ALA and other PUFAs, algae are easy to cultivate, have high photosynthesis efficiency, short reproduction cycle, and offer relatively more flexible production without the need for arable land. Furthermore, abundant microalgae germplasm resources contain numerous genetic resources, which can also be used as chassis organisms and living bioreactors to produce a variety of high value-added products that are beneficial to human and animal nutrition and health. There are several advantages to using microorganisms instead of fish oil as an alternative resource (Table 4).

**TABLE 4:** Comparison of PUFAs production by algae, higher plants and aquatic animals.

Item	Algae	Higher plants	Aquatic animals
Source	Genetic transformation for high-yield strains is relatively mature.	Very few high-yielding species	Fishery resources are limited.
Production	Production throughout the year, not limited by raw materials and origin.	Cultivation is affected by climatic conditions.	The PUFAs varies with the species of fish, season, geography, and fishing time.
	The cultivation and the production quality are relatively easy to control.	The yield and content may be affected by the environment and management.	The living environment of fish is easy to accumulate pollutants.
Harvesting & Extraction	Relatively difficult, and some algae need breaking the wall.	Relatively easy.	Relatively easy.
Purification	Relatively simple. Relatively higher content. Relatively easy enrichment.	Relatively simple. Relatively higher content. Relatively easy enrichment.	Contains a large amount of other saturated and low UFA. Relatively complicated.
Quality	No cholesterol, no fishy smell, no pesticides and heavy metal ion pollution; product characteristics are stable.	No cholesterol, no fishy smell; stable product characteristics. Some crops may have pesticide residues.	Relative higher cholesterol and often has a fishy smell, heavy metals and other environmental pollutants.

### Ways of producing PUFA

Currently, humans rely on fishery products as a source of omega-3 PUFA, which poses a risk of exposure to environmental pollutants such as methylmercury, polychlorinated biphenyls, algal toxins, and other contaminants (Ward and Singh, 2005; Hamilton et al., 2020; Zheng et al., 2023). The consumption of fish and fish oil is less safe due to these pollutants, and the limited fishery resources also make the sustainable supply of omega-3 PUFA challenging (Kris-Etherton et al., 2002; Hamilton et al., 2020). Furthermore, the efficiency of omega-3 PUFA digestion can be affected by the processing methods of aquatic products (Kris-Etherton et al., 2002; Adarme-Vega et al., 2012; Hamilton et al., 2020).

In contrast, microorganisms such as microalgae offer a flexible, efficient, and safe natural synthesis pathway for omega-3 PUFA (Hamilton et al., 2020; Gu et al., 2021). In terms of production costs, compared with fishing and aquaculture to obtain omega-3 PUFA, algae fermentation production still does not have a significant cost advantage. However, with the depletion of fishery resources and the advancement of microalgae processing technology, the cost and product quality of omega-3 PUFA from algae and other microorganisms can eventually capture the market.

For the industrialization of PUFA production from algae, DHA and EPA have been successfully commercialized on a large scale (Borowitzka, 2013; Xue et al., 2013). Currently, several species of microalgae and fungi, including *Schizochytrium*, *Cryptocodinium cohnii*, and diatoms, are being used for fermentation production of PUFA (Ward and Singh, 2005; Marella et al., 2021; Stramarkou et al., 2021; Chi et al., 2022). These studies primarily refer to the fermentation production mode of fungi such as yeast. Many algae can be photoautotrophically cultured in various reactors such as open ponds, and can also be heterotrophically cultured in fermentation tanks. However, commercial algae production mostly relies on autotrophic cultivation, including *Spirulina* sp. and *Chlorella* sp. (Mendes et al., 2022). Although the input cost of photoautotrophic culture is relatively low, the yield per unit cost is also low. Under mixotrophic and heterotrophic conditions, the biomass yield of many algae is even ten times higher than that under autotrophic conditions. If high-density fermentation can be achieved, the unit production cost of some microalgae is lower than that of photoautotrophic culture (Jin et al., 2020; Jin et al., 2021; Ruiz et al., 2022). It is estimated that at a production scale of 1,000 tons/year, the production cost per unit of microalgae biomass production by high-density fermentation method can be lower than that by open pond culture method (Jin et al., 2020). However, it should be noted that the oil content and composition of some algae in heterotrophic culture may not be as commercially valuable as in photoautotrophic culture (Jin et al., 2020). When choosing between photoautotrophic and heterotrophic cultivation, many manufacturers rely on the higher biomass yield per unit of input they can achieve (Ruiz et al., 2022). Therefore, achieving ultra-high-density fermentation is crucial for the commercialization of microalgae. It should be pointed out that, in theory, microalgae fermentation does not require the participation of photosynthesis. If the unnecessary physiological functions and structures of microalgae can be controlled during the proliferation process, or if the synthesis pathway of high-value products such as microalgae PUFA can be introduced into more robust and efficient chassis cells for expression, this could greatly enhance the economic viability of production.

Moreover, the development of sequencing, genetic engineering and bioinformatics technology has significantly contributed to the synthesis of omega-3 PUFA. It has provided essential information for optimizing the enzyme system for algae to synthesize high-value oil (Yang F et al., 2019; Degraeve-Guilbault et al., 2021). The synthetic pathways of PUFAs in algae are relatively well-understood, and many desaturases and elongases in algae or other species have been identified. Additionally, the enzymes present in algae have also provided crucial information for the synthesis pathways of omega-3 PUFA in other species, such as fungi and plants (Rezanka et al., 2017). Compared to the fermentation mode and genetic engineering of yeast and other microorganisms, the tools available for algae still need to be developed (Xue et al., 2013; Xie et al., 2015; Khera and Srivastava, 2022).

Advances in genetic engineering technology are essential for the synthetic biology of algae. However, many algae can only undergo genetic modification, such as RNAi, which cannot be stably inherited (Kugler et al., 2019). Alternatively, high-producing strains can be screened using blind mutagenesis. Nevertheless, if significant breakthroughs occur, many efficient photosynthetic chassis cells could provide a vital platform for the production of PUFA, carotenoids, and other substances. Algae, with their ability to use light energy and cheap carbon sources to produce PUFA, hold great potential for the future. With its high photosynthetic efficiency, algae can be used as chassis cells to transform into a cell factory that can synthesize omega-3 PUFA using solar energy and cheap carbon sources. Thus, genetic engineering technology to transform microbial fermentation for PUFA production is currently an important means to achieve commercialization.

## Pretreatment of lipids extraction from algae

Typically, microalgal biomass obtained through fermentation or other cultures must be harvested prior to lipid extraction. Currently, algae biomass harvesting methods can be divided into four types, flocculation-sedimentation, centrifugation, filtration, and flotation, each with its own scope of application (Wang S et al., 2019; Li et al., 2020). Many materials have been developed to effectively flocculate algae, and various types of collection membranes have been developed to filter and collect algae. However, flocculation, sedimentation, filtration, and flotation are primarily used for the concentration and harvesting of algae with low biomass density, and only most of the medium residue can be removed by separation (Wang S et al., 2019; Li et al., 2020). On the other hand, centrifugation can achieve high-quality concentration and separation of algae, which has been utilized in commercial production and is more suitable for the collection of algae with high cell density (Wang S et al., 2019).

**FIGURE 5:**
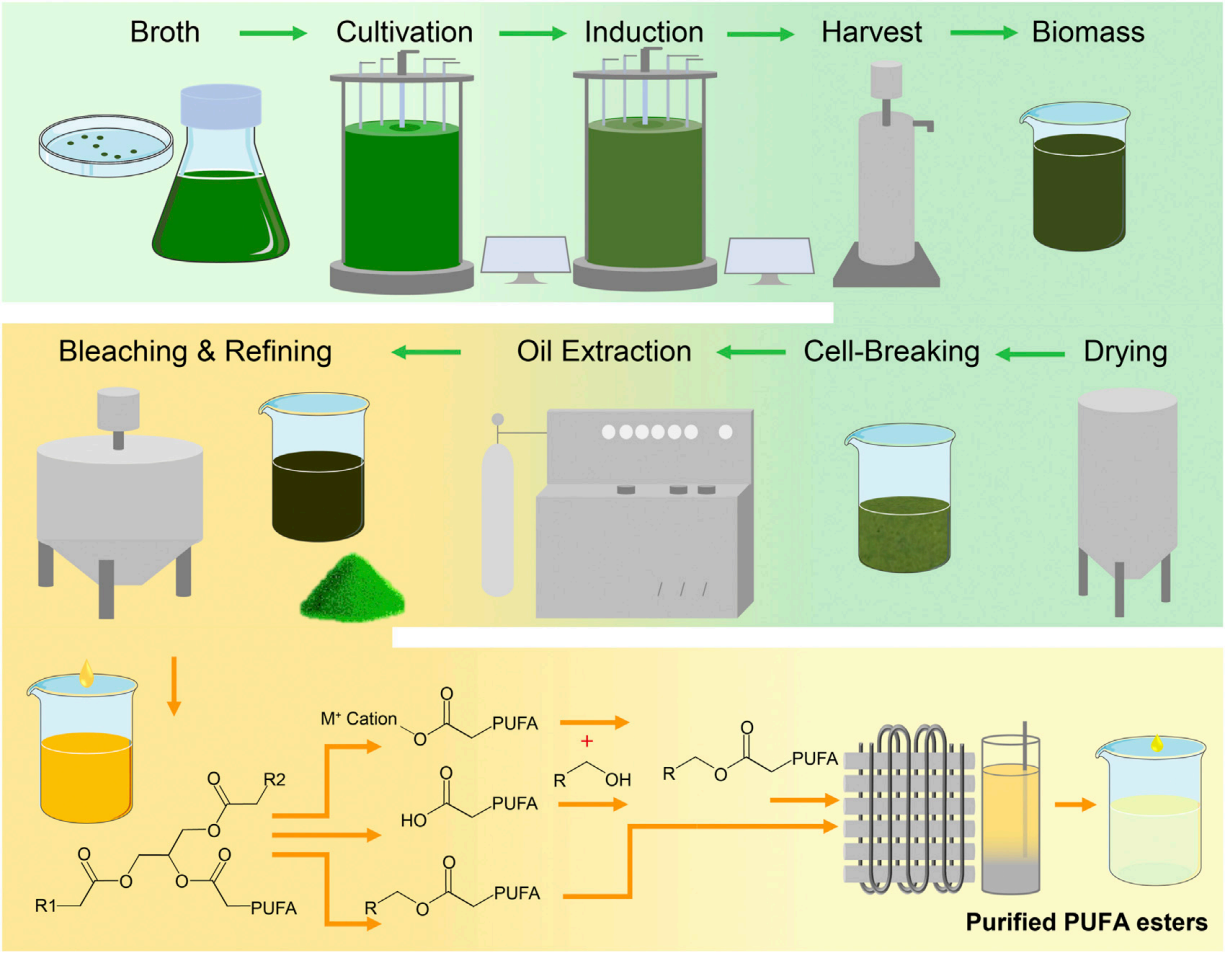
The main production process of high-purity PUFAs.

After being collected by centrifugation, the algae are still wet and need to be dehydrated in order to obtain dried biomass that is stable and easy to storage. While heat drying is a common method, it takes a long time and is not suitable for algae containing high-temperature-sensitive compositions (Hosseinizand et al., 2018). For algae rich in such compositions, like PUFAs, freeze-drying, vacuum drying, and spray drying are more appropriate as they do not involve long-term high-temperature heating. Spray drying involves dispersing wet algae into micro-droplets, which are then exposed to hot air for a very short time, greatly shortening the evaporation time (Zhang et al., 2022). Therefore, spray drying is suitable for large-scale production and is much more efficient than heat drying, freeze drying, and vacuum drying. After the purer algae biomass is collected, oil extraction, impurity removal, and PUFAs purification are required to obtain higher purity algae PUFAs (Ali et al., 2021; Chen W et al., 2021). The process of algae production, oil extraction and PUFAs enrichment is outlined in the Figure 5.

In addition to traditional processes, there is a process in which algae cells are directly converted into fatty acid methyl esters or fatty acid ethyl esters, bypassing the lipids extraction step. Dry or even wet microalgal cells can undergo *in-situ* transesterification to become fatty acid esters (Umamaheswari et al., 2020). Biodiesel production tests using wet *Nannochloropsis gaditana* and *Schizochytium limacinum* biomass have shown that the yield of fatty acid esters in total lipids can reach between 73% and 100% (Kim et al., 2015; Park et al., 2017). Generally, wet microalgae are not suitable for lipid extraction or transesterification reactions due to their moisture content, which prevents non-polar solvents from penetrating into the cells, thereby reducing lipid extraction efficiency (Nagappan et al., 2019). The *in-situ* transesterification reaction is a complex process that involves the reaction between an ionic liquid and the contents of a cell. This process requires breaking down the cell wall to react with the lipid present inside. To enhance the efficiency of this reaction and reduce associated costs, it is essential to conduct a meticulous study of the interaction between the appropriate ionic liquid and the cell components (Ding et al., 2022). However, these processes typically require high temperatures above 100°C, which may cause the degradation of PUFAs. If these limitations can be overcome, it would significantly reduce the production and enrichment process of microalgae PUFA and lower production costs, as illustrated in Figure 5.

## Lipids extraction

Common oil crops such as rapeseed, peanut, soybean, corn, and sunflower can be extracted for lipids using physical pressing and solvent extraction methods. However, due to the powdered or block form of dried algae, physical pressing is not an effective method of extraction. Instead, solvent extraction is generally utilized using substances like n-hexane, chloroform, or supercritical CO_2_. Supplemental techniques such as microwave treatment, mechanical treatment, enzyme treatment, or ultrasonic cell wall breaking can also be applied to improve algae oil extraction (Teo and Idris, 2014).

### Extraction methods

There are various methods available for extracting algae oil, but it is primarily utilized in biodiesel production to reduce costs (Liu et al., 2013). Due to the large number of other components that can be introduced, this method is typically used for extracting products not intended for human and animal consumption. Algae oil extraction methods include chemical solvent extraction, physical wall breaking treatment, supercritical fluid extraction (SFE), and biological extraction (Ali et al., 2021). These methods can be roughly divided into two categories: regular organic solvent extraction and SFE. The advantages, disadvantages, and scope of application of both extraction methods were summarized in Table 5.

**TABLE 5:** Methods for oil extraction from algae.

	Regular organic solvent extraction	Supercritical fluid extraction
Advantage	Relatively high oil yield, simple equipment and easy operation.	Relatively low temperature, avoiding contact with oxygen, high efficiency.
Shortcoming	Toxic, volatile, not conducive to human health and environmental protection.	Expensive and high energy consumption.
Scope of Application	Low-value products, bioenergy.	High-value products, carotenoids, PUFAs.

Organic solvent extraction is widely used in the extraction of algae oil. That is to choose an organic solvent that can dissolve oil, infiltrate the algae and extract the intracellular oil (Ranjan et al., 2010). This method provides high oil yield, simple equipment, and convenient operation. However, the organic solvents used are often toxic and volatile, which can be harmful to human health. While static extraction can extract some substances from the material, its extraction efficiency is low and it takes a long time. To improve the extraction efficiency, dynamic extraction is generally employed, and the solvent is repeatedly flowed through the material (Grierson et al., 2012; Ramluckan et al., 2014; Aravind et al., 2021). For instance, Soxhlet extraction is commonly used to extract the total lipids. This method requires dry biomass with smaller particle size, continuous reflux of the solvent, continuous heating of the equipment, continuous reflux of the evaporated solvent, even continuous reflex with different solvent, making it a highly energy-intensive process that is unsuitable for components that are sensitive to high temperature (Chen W et al., 2020; Aravind et al., 2021).

In a fully enclosed device, SFE is a suitable method for temperature-sensitive products. Carbon dioxide, a non-polar molecule, can be transformed into a supercritical fluid at normal temperature and high pressure. By altering the temperature, pressure, and adding various entrainers, similar polarity molecules in the sample can be extracted (Cooney et al., 2009). Other substances, such as ammonia, ethylene, propane, and propylene, can also be utilized as supercritical fluids (Cooney et al., 2009). In some instances, lipid yields from SFE are comparable to those from Soxhlet extraction (Mercer and Armenta, 2011). Over the last 3 decades, SFE has been utilized to extract high-value compounds from algae, including astaxanthin, β-carotene, lutein, DHA, EPA, ALA, ARA (Solana et al., 2014; Molino et al., 2020). After 5% (w/v) ethanol was used as an entrainer to extract lipids from *Chlorella vulgaris* with supercritical CO_2_, the lipid extraction efficiency increased by 127% (Safi et al., 2014). Lipid extraction yields were also improved by using microwave radiation-assisted supercritical CO_2_ extraction strategies (Dejoye et al., 2011). However, the overall lipid extraction rates they reported, ranging from 47 to 127 mg/g cells, were not significantly higher than those of the physical and chemical methods mentioned above. Compared with Soxhlet extraction, SFE has several advantages in extracting PUFAs from *Scenedesmus* (Solana et al., 2014), *Nannochloropsis* sp. (Jiménez Callejón et al., 2022), *Schizochytrium* sp. (Rodríguez-España et al., 2022).

### Extraction with wall breaking

The extraction of oils from certain algae can be a challenging task as they are protected by cell walls that need to be broken down. The cell wall of algae are complex structures made up of multiple layers, including cellulose and pectin, which are quite rigid and can impede the extraction of target compounds (Brennan and Owende, 2010). Currently, the main methods for breaking down algae cell walls are physical methods that mechanically destroy the cell wall, as well as biochemical methods that use enzymes or chemical agents to hydrolyze and degrade the cell wall. However, the appropriate cell wall breaking method largely depends on the unique biology and cell wall characteristics of the algae (Praveenkumar et al., 2015). Additionally, the efficiency of these methods is affected by operating conditions such as temperature, pressure, biomass (concentration, wet and dry state, growth stage), and scale (Rawat et al., 2011). If the synthesis of cell walls can be controlled through genetic engineering, the barrier to substance extraction can be eliminated, significantly reducing the cost of material processing, and also correspondingly increasing the utilization rate of nutrients in the culture medium. Therefore, creating excellent chassis cells and controlling the synthesis of cell walls is the most ideal solution.

#### Physical wall-breaking method

The physical wall-breaking method involves applying mechanical external force or non-mechanical physical methods to break up cells, which include homogenization, high-pressure homogenization, ultrasonic crushing, microwave crushing, electroporation, autoclave, repeated freezing and thawing (Madany et al., 2021).

The homogenization utilizes mechanical external force, such as grinding, to break the wall. When crushing *Chlorococcum* sp. by grinding, the average cell crushing rate reaches 17.5% (Halim et al., 2012). Homogenization is only appropriate when the product is easily separable. Although it is economical and environmentally friendly, and does not require mixing with other ingredients, it is not efficient for algae with stronger cell walls (Madany et al., 2021).

The high-pressure homogenization method uses high-speed impact and strong shearing under high pressure to break up cells. When treating *Chlorococcum* sp. with 85 MPa homogenization, the cell disruption rate reaches 73.8% (Halim et al., 2012). The crushing effect of the high-pressure homogenization method is closely related to the composition of the algae cell wall. This method usually has a high breaking rate and has little effect on fatty acid profile, but is energy intensive (Magpusao et al., 2022).

Ultrasonication is suitable for most algal cells and generates dense microbubbles in liquid media, resulting in strong cavitation effects when high-power ultrasound (typically exceeding 20 kHz) is used (Valizadeh Derakhshan et al., 2014). Proper ultrasonic treatment can even double the oil extraction rate from *Chlorella* (Ellison et al., 2019). Ultrasonic method is non-polluting and has a high wall-breaking rate, but the local temperature in the system may rise sharply, and its effect on the quality of the extracted oil is not yet clear.

The microwave crushing method employs microwave heating to rapidly increase the intracellular temperature, which results in the vaporization of liquid water and the generation of high pressure, ultimately breaking through the membrane-wall structure of the cell to create small holes (Lee et al., 2010). Although this method is fast and pollution-free, the sharp increase in temperature may cause denaturation of the active substance.

Electroporation generates minimal cell debris and can even promote cell growth and regeneration. It is also possible to extract intracellular lipids into the supernatant. However, the extraction rate is lower compared to chemical lysis or mechanical decomposition (Eleršek et al., 2020).

Autoclaving involves the use of high temperature and pressure to increase the diffusion of substances, allowing them to be released from cells (Zhou et al., 2020). However, it may cause side reactions in some substances and lead to changes in lipids. This method is typically used in bioenergy production (Sivaramakrishnan and Incharoensakdi, 2019).

In contrast, the repeated freezing and thawing method produces ice particles within the cells, causing the salt concentration of the remaining cytosol to increase, leading to cells swelling and breakage. This technique is simple to perform and does not damage heat-sensitive substances, making it suitable for the extraction of high-value substances, making it suitable for high-value product extraction using wall-broken extraction. However, it consumes more energy compared to other methods.

#### Biochemical wall-breaking method

The biochemical wall-breaking method involves adding biological enzymes or chemical reagents to assist in degrading cell wall. This approach is commonly used in conjunction with physical methods. The most prevalent methods are the osmotic shock method, solvent lysis method, ionic liquid lysis method, switchable polar solvent lysis method, and biological enzyme preparation digestion method.

The acid-heat method involves treating algae with acid to degrade the cell walls. Mixing *Chlorococcum* sp. with 8% sulfuric acid and heating the mixture at 160°C for 45 min achieved an algae cell wall breaking rate of 33.2% (Halim et al., 2012). Dilute H_2_SO_4_ can significantly affects the degradation of cell wall, even in the absence of any polar organic solvent such as methanol. However, it should also be noted that this sulfuric acid process can be efficiently applied to wet algal biomass, but appropriate material and facility costs should be addressed (Park et al., 2014).

Chemiosmosis involves using the difference in osmotic pressure between the inside and outside of algae cells in a hypertonic solution to break down the cells. Although this method is mild, it usually takes a long time, and the high osmotic pressure causes the cell contents to be squeezed out spontaneously. A low lipid extraction rate was obtained from wet algal biomass of *C. vulgaris* with 1 L of phosphorus hydrate P(CH_2_OH)_4_Cl (Olkiewicz et al., 2015).

Solvent lysis, ionic liquid lysis, and switchable polarity solvent lysis are based on the properties of the cell wall, where specific solvents or chemical substances are added to degrade the cell wall. The most common solvent lysis methods include the Bligh and Dyer method, Folch method, and Soxhlet extraction, which simultaneously achieve cell wall lysis and oil extraction. The ionic liquid lysis supplemented by physical treatment can significantly enhance the extraction of fatty acids. Switchable polarity solvents have unique chemical properties that can rearrange the lipid bilayer structure in the cell membrane, resulting in higher extraction efficiency than solvent lysis.

The enzymatic method utilizes hydrolytic enzymes to degrade algae cells. This method has a high rate of wall breaking, mild conditions, and does not cause denaturation of intracellular substances. However, the use of enzyme preparations also increases the difficulty of separating and purifying later products. Enzymatic hydrolysis of *Chlorella vulgaris* is superior to other physical breaking methods (Zheng et al., 2011). When comparing the effects of various cell disruption methods on lipid production in *Schizochytrium* sp., pretreatment with lysozyme and cellulase results in the highest extraction rate and highest DHA content (Hac İsa et al., 2022).

## Decolorization and impurity removal

Crude or unrefined bio-oil often contains various other fat-soluble molecules, such as photosynthetic pigments, which are valuable nutritionally. However, as a commodity oil, these compositions not only impact the color and appearance of the oil but also reduce the stability of the oil quality, leading to oxidation or deterioration of the oil (Rodríguez-España et al., 2022). Therefore, it is crucial to remove unnecessary components such as pigments to improve the oil’s stability and quality.

Algae lipids generally contain a certain amount of carotenoids, which are fat-soluble. It has been found that carotenoids are typically synthesized in cells and stored in lipid droplets in a fat-soluble form to accumulate a substantial amount of astaxanthin, which requires synthesizing the necessary amount of lipid droplets (Collins et al., 2011; Han et al., 2013). Although there are several separation methods to separate carotenoids from lipids, such as silica gel column chromatography, central partition chromatography, and thin-layer chromatography (Sun et al., 2022), the separation cost is relatively high. Additionally, carotenoids and lipids can be separated by screening solvents and using the polarity difference (Lourenço-Lopes et al., 2020; Sun et al., 2022). Separation can improve the output value of production if the product value exceeds the increased process cost. Some algae can also synthesize carotenoids when accumulating large amounts of long chain PUFA under special conditions. For example, *Chaetoceros gracilis* (Tachihana et al., 2020), *Phaeodactylum tricornutum* (Derwenskus et al., 2020)*, Nitzschia* sp (Ma et al., 2023) can simultaneously accumulate EPA and fucoxanthin, while *Nannochloropsis gaditana* (Menegol et al., 2019) can simultaneously accumulate EPA and carotenoids, while *Aurantiochytrium* sp (Yin et al., 2023) can simultaneously accumulate DHA, astaxanthin, and β-carotenoids. However, according to the reported content, in order to simultaneously accumulate both types of high-value products in large quantities, further in-depth research is still required.

Adsorbents commonly used for crude oil decolorization include various products such as activated clay and activated carbon. Among them, activated clay is widely used for decolorization in the oil industry. Activated clay is inexpensive, easy to obtain and prepare, and has a strong absorption capacity for chlorophyll, carotenoids and their derivatives, hydroxyl-containing polar molecules, free fatty acids, and colloidal substances (Kreps et al., 2014). For example, it effectively decolorizes crude oil from corn (Liu and Zheng, 2014) and *Scenedesmus* sp. (Chen W et al., 2021).

Activated clay is a commonly used adsorbent for crude oil decolorization, which can be obtained from various sources. However, activated clay from different production areas may have varying components, leading to differences in their decolorization performance. Therefore, modifications can be made to activated clay to enhance its adsorption and decolorization capabilities. Activated clay is preferred over other decolorization materials, such as activated carbon, activated fuller’s earth and attapulgite clay, making it a more suitable choice (Liu and Chen, 2012). Not only can activated clay effectively decolorize crudely extracted biodiesel, but it can also remove most peroxides (Su et al., 2014). For example, commercial montmorillonite clay from Leping, Jiangxi, China (Yang M et al., 2019), and upper Eocene sedimentary clay from Kairouan, Tunisia (Eloussaief et al., 2020) can be modified through proper treatment, greatly improving the adsorption capacity of pigment molecules in oil. Moreover, some activated clay used for oil decolorization can be regenerated by calcination and other treatments. Recycled activated clay can then be used again, reducing the cost of decolorization (Bachmann et al., 2020). To optimize the decolorization process, it is necessary to screen the activated clay with the best decolorization performance for the target oil. Appropriate improvement of the activated clay processing technology can also improve the decolorization performance. Finally, optimizing the decolorization conditions can lead to activated clay becoming an ideal tool for the decolorization of refined oil and algae oil (Chen et al., 2012).

## Enrichment of PUFAs

Many algae have been approved as a new food resource and are already being incorporated into everyday meals, with related food products already on the market. Some people may need to supplement their omega-3 PUFA-rich intake through consuming foods and edible oils rich in these fatty acids, while others may need to turn to drugs, health products, or even milk powder. To manufacture these products, omega-3 PUFA raw materials with a single component and defined content are required. Algae oil, which is rich in omega-3 PUFAs, can be used as a raw material for these products. However, to meet the desired product characteristics when used in health products, pharmaceuticals, or auxiliary medicines, PUFAs in algae oil must be purified (Li X et al., 2019; Wei and Wang, 2020). For instance, several omega-3 PUFAs prescription drugs approved by the U.S. Food and Drug Administration for the prevention and treatment of high triglycerides require even higher purity (Hoy and Keating, 2009; Blair and Dhillon, 2014; Kim and McCormack, 2014).

### Hydrolysis of lipids

Fatty acid hydrolysis can hydrolyze and separate ester-forming fatty acids, which can then be further separated and utilized based on their different properties.

#### Saponification-esterification method

PUFAs can be separated and concentrated by taking advantage of the difference in the solubility of fatty acid metal salts in certain organic solvents. To obtain refined oil with higher PUFAs content, lipids can be saponified under heating conditions using a specific ratio of ethanol and NaOH. The saponified mixture can then be filtered to obtain saponified liquid and solid particles, followed by esterification of the saponified compositions with dilute H_2_SO_4_.

#### Non-catalytic hydrolysis method

Transesterification of triglycerides to form fatty acid methyl esters or fatty acid ethyl esters can be achieved directly under high temperature and pressure (Felix et al., 2019), through the Thermal-Fenton mechanism (Yew et al., 2021), or in supercritical methanol conditions (Mohamadzadeh Shirazi et al., 2017). However, this process is mainly used in biodiesel production, which results in impurities and may easily damage PUFAs.

#### Chemical catalytic hydrolysis method

Acid catalysis, saponification-acidolysis, and high-pressure hydrolysis are common methods used in the oleochemical industry for lipid hydrolysis. The acid-catalyzed and saponification-acidification methods are cost-effective and have a hydrolysis rate of over 90%. However, they produce large amounts of waste acid or alkali (Zhuang et al., 2012; Asikainen et al., 2015). High-pressure steam hydrolysis is an effective alternative but requires higher equipment and operating costs. Generally, these methods are carried out at higher temperatures, particularly the high-pressure hydrolysis method (Zarli, 2020).

Chemical catalysts can be categorized into homogeneous and heterogeneous catalysts, which can be further subdivided into alkaline and acid catalysts (Neag et al., 2023). Commonly used homogeneous catalysts include NaOH, CH_3_ONa, KOH, concentrated HCl, or H_2_SO_4_ (Neag et al., 2023). Heterogeneous catalytic processes are suitable for raw materials containing more polar components, such as CaO/MgO, KF/CaO, magnetic nano-scale KOH/Fe_2_O3-Al_2_O_3_ (Kazemifard et al., 2019), CaMgO/Al_2_O_3_, zirconia (ZrO_2_), titanium oxide (TiO_2_), zeolites, ion exchange resins (Hidalgo et al., 2013), and algae carbon-based solid acid catalysts (Cao et al., 2021).

#### Lipase-catalyzed method

Enzymatic enrichment utilizes the selective specificity of lipase, an enzyme that catalyze various reactions such as hydrolysis, acidolysis, alcoholysis, transesterification and reverse synthesis of esters of triacylglycerides and other water-insoluble esters. The main reactions involved in enzymatic enrichment are hydrolysis, transesterification and esterification.

##### Enzymatic hydrolysis

The enzymatic hydrolysis method, which employs lipase for breaking down oil into fatty acids and glycerol, can be carried out at mild conditions without the need for steam boiling or pressurization. The reaction can be carried out at room temperature or slightly higher, and does not produce a large amount of waste acid or waste alkali. Lipase is a carboxyl ester hydrolase that can hydrolyze the α and β bonds of glycerides into fatty acids and glycerol and synthesize glycerol esters from fatty acids and glycerol. Depending on the characteristics of different lipases and the selected reaction conditions, both hydrolysis and transesterification reactions can be operated, as well as esterification of certain fatty acids. By undergoing hydrolysis, transesterification, and esterification reactions, lipases can produce high concentrations of specific fatty acids, which can be subsequently isolated and enriched by other methods (Akanbi et al., 2013). Lipase-catalyzed hydrolysis has proven successful in enriching EPA and DHA in oils from fish (Pan et al., 2012; Valverde et al., 2013; Valverde et al., 2014), *Isochrysis galbana*, *Chaetocerous calcitran*, *Chlorella marina*, and *Tetraselmis gracillus* (Jacob and Mathew, 2017), and ALA in flaxseed oil (Rupani et al., 2012; Zou et al., 2020). Hence, lipase can be an efficient and environmentally friendly tool to enrich fatty acids. The hydrolysis rate of some lipases for olive oil, soybean oil, rice bran oil, linseed oil, perilla seed oil, and rapeseed oil can reach 35%–100% (Zhao et al., 2022), and the hydrolysis rate of oil to *Scenedesmus* sp. can reach 66% (Chen W et al., 2021).

##### Enzymatic esterification method

Lipase selectively catalyzes the reaction of UFAs and alcohols to produce esters, or between saturated fatty acids and alcohols to generate esters, thereby enriching UFAs (Jithu Paul, 2019).

##### Enzymatic transesterification method

It involves the catalytic exchange of acyl groups between glycerides and free fatty acids, alcohols, or other esters under the catalysis of lipase. These reactions are referred to as acidolysis, alcoholysis and transesterification reactions, respectively. This method is particularly suitable for the preparation of high concentration PUFAs or PUFA methyl esters (ethyl esters) (Farmani et al., 2007; Castejón and Señoráns, 2020). During the transesterification process, lipase preferentially acts on saturated and monounsaturated chains, thereby substituting PUFAs into glycerides to achieve enrichment.

Chemical methods for enriching PUFAs usually require derivatization of raw oil to form PUFAs or their methyl and ethyl esters, which then need to be combined with other separation methods to achieve a good enrichment effect. Additionally, these methods often require further conversion into a glyceride form that can be easily absorbed by the human body. Physical and chemical methods also involve the use of a large amount of organic reagents, which can be harmful to health and must to be completely removed. In contrast, the lipase hydrolysis method only requires a single-step reaction under mild conditions and does not require a large amount of organic reagents or additional pretreatment. This method can enrich PUFAs on glycerides, and the hydrolysis rate of these fatty acid glycerides is faster than the methyl or ethyl ester enriched by physical and chemical methods (Yang et al., 1989). Therefore, the hydrolysis method is more suitable for human digestion and absorption. The immobilized lipase can still maintain 60% of its initial activity even after 10 reaction cycles (Liu et al., 2018). However, to separate the target components, it is often necessary to combine other methods. Lipase with high specificity must be extensively screened, which can increase the cost. To enhance the functional specificity of lipase, computer-aided design can be employed (Moharana and Rao, 2020).

### Purification of PUFAs

Various methods are available for the separation and purification methods of PUFAs, such as low-temperature crystallization, urea inclusion, silver ion complexation, molecular distillation methods. The advantages and disadvantages of these methods are summarized in Table 6.

**TABLE 6:** ALA enrichment methods.

	Advantages	Disadvantages
Solvent Extraction	Simple operation	Low purity
Silver Ion Complexation	Good separation effect.	Expensive. Heavy metal pollution.
	High product purity	
Molecular Distillation	No foreign substances’ introduction.	Need high vacuum.
	Temperature is far below the boiling point.	High energy consumption.
	Short heating time.	Low purity of the product.
	Fit for continuous production.	Not ideal for the separation of fatty acids with similar molecular weights.
Low-temperature Crystallization	Cheap equipment, easy to operate, and can effectively protect active ingredients	A large amount of organic solvent needs to be recovered, and the separation efficiency is not high.
Urea Complexation	Simple process, low cost, and the active ingredients protective.	Difficulty separating fatty acids with similar degrees of unsaturation.

#### Ag^+^ complexation method

Silver ions have the ability to form polar complexes with carbon-carbon double bonds, and the more double bonds there are, the more Ag^+^ combined, the stronger the complexation, and the more stable the complex. The silver ion complexation method is typically categorized into the AgNO_3_ solution direct complexation method, AgNO_3_-silica gel column chromatography, AgNO_3_ membrane separation and adsorption method, etc. For instance, the Ag^+^ complexation method has been used to enrich omega-3 PUFA from *Desmodesmus* sp. oil, leading to an increase in the ALA content from 24% to 92% (Nagappan and Kumar Verma, 2018). These methods have high enrichment efficiency and product purity, but the cost of Ag^+^ is high and recovering it in large quantities is challenging. Moreover, after saponification, Ag^+^ needs to be eluted with organic reagents which may cause heavy metal pollution in the product and residues in organic eluents. These drawbacks have limited the widespread production and application of these methods.

#### Molecular distillation method

Molecular distillation is a method used to separate substances based on the difference in mean free path of the molecular thermal motion of each component in the mixture. The molecular mean free path is determined by the molecular diameter, system pressure, and temperature. Under specific temperature and pressure conditions, fatty acids with high degrees of unsaturation have a short molecular mean free path, resulting in a slower evaporation rate. Multistage distillation can efficiently separate different components (Zhang et al., 2013; He et al., 2020). Molecular distillation doesn’t require the introduction of foreign substances, does not need to be heated to the boiling point, has a short heating time, and can be produced continuously. However, it must be performed under high vacuum, has high energy consumption, and may yield products with low purity. Thus, when separating fatty acids with similar molecular mass, molecular distillation is often combined with other separation methods. For example, continuous four stages of molecular distillation can increase the mass fraction of ALA in silkworm chrysalis oil from 54.49% to 99.28% (Liu et al., 2018), and enrich ω-3 PUFAs in *Schizochytrium limacinum* oil to 92.98% (Zhang et al., 2013).

#### Low-temperature crystallization method

**FIGURE 6:**
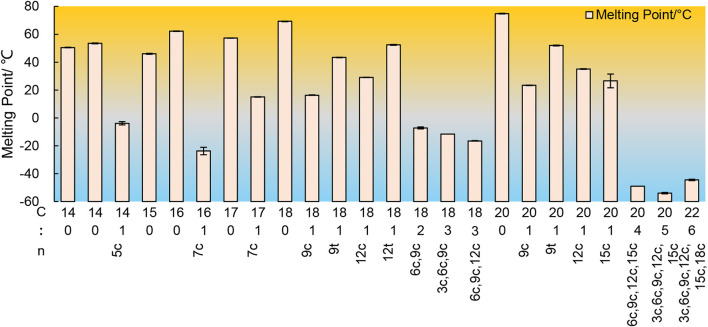
The melting points of some free fatty acids.

The low-temperature crystallization method is primarily based on the difference in freezing point and solubility at low temperature conditions for the purpose of separation and purification. The melting point of saturated fatty acids is usually higher than that of UFAs, meaning that the solidification temperature of fatty acids with more unsaturated bonds is lower. Additionally, temperature also affects the solubility of fatty acids in organic solvents. As a general rule, fatty acids with longer carbon chains exhibit lower solubility, while fatty acids with more double bonds have higher solubility in organic solvents, as shown in Figure 6 (Knothe and Dunn, 2009). Therefore, low-temperature crystallization permits saturated fatty acids in fatty acids to form a solid phase while UFAs can dissolve in organic solvents, thereby enriching UFAs in the remaining liquid phase. The separation technique involves two steps, namely oil crystallization and solid-liquid phase separation. It can be divided into dry fractionation, surfactant fractionation, and solvent fractionation.

Dry fractionation refers to the transesterification of crude oil under alkaline conditions to produce fatty acid methyl ester (ethyl ester), which is then cooled to a specific temperature without the addition of any organic solvent, allowing the solid components in the oil to crystallize first (Soleimanian et al., 2015). The solid and liquid phases are then separated by centrifugal filtration or other means. However, methanol-based alcoholysis is toxic and hazardous to human health, and thus many studies employ ethanol. Additionally, fatty acid ethyl ester is not easily hydrolyzed by pancreatic lipase, which is not beneficial for human absorption (Sigurgisladottir et al., 1992).

Surfactant fractionation entails freezing crude oil for natural crystallization, followed by the addition of a specific proportion of surfactant and electrolyte solution to emulsify the crystallized solid oil and suspend it in the water phase, thus increasing the separation efficiency of crystallized solid oil (Salminen et al., 2014; Islam et al., 2020). This method is more efficient than the dry method, but many surfactants cannot be utilized in food processing.

Solvent fractionation involves saponifying crude oil into free fatty acids, selecting an appropriate organic solvent to dissolve in a specific proportion, and refrigerating it at low temperature. Subsequently, large amounts of fatty acids with low degrees of unsaturation are crystallized and separated by filtration. Using the low-temperature crystallization method, the content of ALA in silkworm chrysalis oil increased from 15% to 47.6% (Wang et al., 2013), and the content of UFAs in the oil of *Scenedesmus* sp. can be increased to more than 90%, of which ALA can reach 63% (Chen W et al., 2021).

#### Urea inclusion

Urea can form clathrates with aliphatic compounds, specifically straight-chain compounds containing more than 4 carbon atoms (Domart et al., 1955; Hollingsworth et al., 1996). Urea inclusion has long been recognized as an effective method for enriching PUFA, as indicated in Table 7. The urea clathrate is based on the linear aliphatic compound as the axis, where there is a weaker van der Waals bond between urea and the linear aliphatic compound. Urea molecules spiral up around the axis in a counterclockwise direction through hydrogen bonding, tightly enclosing it (González-Fernández et al., 2020). During the crystallization process, urea forms relatively stable crystal inclusion complexes with saturated fatty acids or monounsaturated fatty acids. However, polyvalent unsaturated fatty acids are difficult to include due to their multiple double bonds and curved carbon chains, which have a specific spatial configuration (Shen et al., 2018). A variety of urea + X inclusion methods derived from the improvements can be used to design polymer-urea inclusion complexes more accurately. These methods make it easier to include straight chain molecules while making it more challenging to clathrate curved polymer molecules (Shen et al., 2018). The entire process of the urea inclusion method is simple to operate, and the necessary raw materials are readily available and inexpensive. This method is worth considering not only for large-scale application but also as it has a strong experimental foundation for reference.

**TABLE 7:** The effects of urea inclusion for PUFAs enrichment.

Target	Effect	Reference
LA in *Clarias macrocephalus* oil	Enriched from 18.4% to 47%	Zainuddin *et al.* (2011)
ALA in comfrey oil	Enriched to 99.30%	Han *et al.* (2004)
ALA in Kiwi Seed Oil	Enriched from 61.82% to 87.2%	Wu *et al.* (2005)
ALA in Silkworm Chrysalis Oil	Enriched from 27.99% to 70.28%	Zhang *et al.* (2011)
ALA in Flaxseed Oil	Enriched to 78.6%	Cheng *et al.* (2017)
GLA in comfrey oil	Enriched to 91%	Spurvey and Shahidi (2000)
DHA in waste oil by-products	Enriched from 25.5% to 59.7%	Wayan Suriani and Komansilan (2019)
PUFAs in carp fish oil	Enriched to 88.9%	Crexi *et al.* (2012)
PUFAs in Seal Oil	Enriched to 71.35%	Zheng *et al.* (2018)
PUFAs in Refined Salmon Oil	Enriched from 38.4% to 96.99%	Dovale-Rosabal *et al.* (2019)

## Outlook

Essential PUFAs have numerous health and medical benefits. As people’s quality of life improves, their awareness of health protection increases, and they pay more attention to nutrition and health in their diet. Providing high-quality PUFAs for the population can help enhance their health and quality of life, and indirectly reduce medical expenses for society. Therefore, providing high-quality PUFAs for the general population has both significant social significance and good market prospects. However, there are few sources of PUFAs-rich oils on the market, and the production of PUFAs-rich algae oils is still at the laboratory-scale stage. Many algae that can produce high PUFAs are still in the verification stage and may be listed as new resource foods in the future. Thus, it is necessary to explore the technical model of large-scale production of these high-yield PUFAs algae, master practical industrial technology means, lay a technical foundation for the industrialization of algae edible oil, and promote algae resources to the market.

In the field of bioenergy research, the production of algae oil is currently facing a bottleneck due to high production costs. As a result, commercial promotion has become challenging, and progress in commercial applications have been slow. While the industrialization of high-value algal oils such as DHA and astaxanthin has already been successfully realized, producing high-value PUFAs from algae could potentially solve the cost problem of algae biodiesel by utilizing the separated by-product of saturated fatty acid as the raw material for algae biodiesel. Algae bioenergy currently faces a cost bottleneck that is difficult to overcome, but focusing on the development and utilization of high-value products could make it more economically feasible.

An analysis of the research hotspots and development direction of algae PUFAs production indicates that while there is considerable emphasis on the accumulation of algae PUFAs, downstream processes such as extraction, decolorization, impurity removal, enrichment, and purification require more attention. This work provides a systematic description of the necessary process methods for converting algae biomass to enriched PUFAs, and offers guidance for the production of omega fatty acids using algae.

## Abbreviation

AdA, Adrenic acid; ARA, Arachidonic acid; ALA, Alpha-linolenic acid; DGLA, Dihomo-gamma-linolenic acid; DHA, Docosahexaenoic acid (Cervonic acid); DPA, Docosapentaenoic acid (Clupanodonic acid); EDA, Eicosadienoic acid; EPA, Eicosapentaenoic acid (Timnodonic acid); ETA, Eicosatetraenoic acid; ETE, Eicosatrienoic acid; GLA, Gamma-linolenic acid; HPA, Heneicosapentaenoic acid; HTA, Hexadecatrienoic acid; LA, Linoleic acid; OA, Oleic acid; PUFA(s), Polyunsaturated Fatty Acid(s); SDA, Stearidonic acid.

## References

[B1] Abedin L. Lien E. L. Vingrys A. J. Sinclair A. J. (1999). The effects of dietary alpha-linolenic acid compared with docosahexaenoic acid on brain, retina, liver, and heart in the Guinea pig. Lipids 34, 475–482. 10.1007/s11745-999-0387-3 10380119

[B2] Adarme-Vega T. C. Lim D. K. Y. Timmins M. Vernen F. Li Y. Schenk P. M. (2012). Microalgal biofactories: A promising approach towards sustainable omega-3 fatty acid production. Microb. Cell Factories 11, 96. 10.1186/1475-2859-11-96 PMC3465194 22830315

[B3] Aguillón-Páez Y. J. Romero L. A. Diaz G. J. (2020). Effect of full-fat sunflower or flaxseed seeds dietary inclusion on performance, egg yolk fatty acid profile and egg quality in laying hens. Anim. Nutr. 6, 179–184. 10.1016/j.aninu.2019.12.005 32542198 PMC7283366

[B4] Akanbi T. O. Adcock J. L. Barrow C. J. (2013). Selective concentration of EPA and DHA using *Thermomyces lanuginosus* lipase is due to fatty acid selectivity and not regioselectivity. Food Chem. 138, 615–620. 10.1016/j.foodchem.2012.11.007 23265531

[B5] Ali Z. Subeshan B. Alam M. A. Asmatulu E. Xu J. (2021). Recent progress in extraction/transesterification techniques for the recovery of oil from algae biomass. Biomass Convers. Biorefinery 13, 2553–2569. 10.1007/s13399-021-01326-y

[B6] Amorim M. L. Soares J. Coimbra J. Leite M. O. Albino L. F. T. Martins M. A. (2020). Microalgae proteins: Production, separation, isolation, quantification, and application in food and feed. Crit. Rev. food Sci. Nutr. 61, 1976–2002. 10.1080/10408398.2020.1768046 32462889

[B7] Anzhany D. DespalToharmat T. Nuraina N. Hamidah A. N. Rofiah N. (2021). Effect of different altitudes on milk fatty acid and conjugated linoleic acid (CLA) profiles. IOP Conf. Ser. Earth Environ. Sci. 667, 012102. 10.1088/1755-1315/667/1/012102

[B8] Aravind S. Barik D. Ragupathi P. Vignesh G. (2021). Investigation on algae oil extraction from algae Spirogyra by Soxhlet extraction method. Mater. Today Proc. 43, 308–313. 10.1016/j.matpr.2020.11.668

[B9] Asikainen M. Munter T. Linnekoski J. (2015). Conversion of polar and non-polar algae oil lipids to fatty acid methyl esters with solid acid catalysts – a model compound study. Bioresour. Technol. 191, 300–305. 10.1016/j.biortech.2015.05.001 26004380

[B10] Aşkın B. Kaya Y. (2020). Effect of deep frying process on the quality of the refined oleic/linoleic sunflower seed oil and olive oil. J. Food Sci. Technol. 57, 4716–4725. 10.1007/s13197-020-04655-4 33087982 PMC7550478

[B11] Ayerza R. (1995). Oil content and fatty acid composition of chia (*Salvia hispanica* L.) from five northwestern locations in Argentina. J. Am. Oil Chemists’ Soc. 72, 1079–1081. 10.1007/BF02660727

[B12] Bachmann S. A. L. Valle R. d. C. S. C. Vegini A. A. Tavares L. B. B. (2020). Determination of optimum conditions for thermal regeneration and characterization of a spent bleaching Earth. J. Environ. Chem. Eng. 8, 103503. 10.1016/j.jece.2019.103503

[B13] Becker W. (2003a). Microalgae for aquaculture: The nutritional value of microalgae for aquaculture. Handb. Microalgal Cult. 2003, 380–391. 10.1002/9780470995280.ch21

[B14] Becker W. (2003b). Microalgae in human and animal nutrition. Handb. Microalgal Cult. 2003, 312–351. 10.1002/9780470995280.ch18

[B15] Bell M. V. Batty R. S. Dick J. R. Fretwell K. Navarro J. C. Sargent J. R. (1995). Dietary deficiency of docosahexaenoic acid impairs vision at low light intensities in juvenile herring (*Clupea harengus* L.). Lipids 30, 443–449. 10.1007/Bf02536303 7637565

[B16] Blair H. A. Dhillon S. (2014). Omega-3 carboxylic acids (Epanova®): A review of its use in patients with severe hypertriglyceridemia. Am. J. Cardiovasc. Drugs 14, 393–400. 10.1007/s40256-014-0090-3 25234378

[B17] Borowitzka M. A. (2013). High-value products from microalgae—Their development and commercialisation. J. Appl. Phycol. 25, 743–756. 10.1007/s10811-013-9983-9

[B18] Boskabady M. H. Kaveh M. Shakeri F. Mohammadian Roshan N. Rezaee R. (2019). Alpha-linolenic acid ameliorates bronchial asthma features in ovalbumin-sensitized rats. J. Pharm. Pharmacol. 71, 1089–1099. 10.1111/jphp.13094 30993723

[B19] Brenna J. T. Salem N. Sinclair A. J. Cunnane S. C. (2009). α-Linolenic acid supplementation and conversion to n-3 long-chain polyunsaturated fatty acids in humans. Prostagl. Leukot. Essent. Fat. Acids 80, 85–91. 10.1016/j.plefa.2009.01.004 19269799

[B20] Brennan L. Owende P. (2010). Biofuels from microalgae—a review of technologies for production, processing, and extractions of biofuels and co-products. Renew. Sustain. Energy Rev. 14, 557–577. 10.1016/j.rser.2009.10.009

[B21] Brett M. Müller-Navarra D. (1997). The role of highly unsaturated fatty acids in aquatic foodweb processes. Freshw. Biol. 38, 483–499. 10.1046/j.1365-2427.1997.00220.x

[B22] Calder P. C. Grimble R. (2002). Polyunsaturated fatty acids, inflammation and immunity. Eur. J. Clin. Nutr. 56 (3), S14–S19. 10.1038/sj.ejcn.1601478 12142955

[B23] Cao M. Peng L. Xie Q. Xing K. Lu M. Ji J. (2021). Sulfonated *Sargassum horneri* carbon as solid acid catalyst to produce biodiesel via esterification. Bioresour. Technol. 324, 124614. 10.1016/j.biortech.2020.124614 33434876

[B24] Capek L. Uhliarikova I. Kostalova Z. Hindakova A. Capek P. (2023). Structural properties of the extracellular biopolymer (beta-D-xylo-alpha-D-mannan) produced by the green microalga *Gloeocystis vesiculosa* Nageli. Carbohydr. Res. 525, 108766. 10.1016/j.carres.2023.108766 36812847

[B25] Castejón N. Señoráns F. J. (2020). Enzymatic modification to produce health-promoting lipids from fish oil, algae and other new omega-3 sources: A review. New Biotechnol. 57, 45–54. 10.1016/j.nbt.2020.02.006 32224214

[B26] Chauhan A. S. Patel A. K. Chen C.-W. Chang J.-S. Michaud P. Dong C.-D. (2023). Enhanced production of high-value polyunsaturated fatty acids (PUFAs) from potential thraustochytrid *Aurantiochytrium* sp. Bioresour. Technol. 370, 128536. 10.1016/j.biortech.2022.128536 36581232

[B27] Cheah Y. T. Ng B. W. Tan T. L. Chia Z. S. Chan D. J. C. (2022). Biomass and eicosapentaenoic acid production from *Amphora* sp. under different environmental and nutritional conditions. Biotechnol. Appl. Biochem. 2022, 2379. 10.1002/bab.2379 35767864

[B28] Chen H. Li K. Xue C. Wang Q. (2021). A novel method for non-invasive estimation of primary productivity in aquatic ecosystems using a chlorophyll fluorescence-induced dynamic curve. Front. Microbiol. 12, 682250. 10.3389/fmicb.2021.682250 34194414 PMC8236984

[B29] Chen H. Li T. Wang Q. (2019). Ten years of algal biofuel and bioproducts: Gains and pains. Planta 249, 195–219. 10.1007/s00425-018-3066-8 30603791

[B30] Chen H. Wang Q. (2022). Microalgae-based green bio-manufacturing—how far from us. Front. Microbiol. 13, 832097. 10.3389/fmicb.2022.832097 35250947 PMC8891535

[B31] Chen H. Wang Q. (2020). Microalgae-based nitrogen bioremediation. Algal Res. 46, 101775. 10.1016/j.algal.2019.101775

[B32] Chen H. Wang Q. (2021). Regulatory mechanisms of lipid biosynthesis in microalgae. Biol. Rev. 96, 2373–2391. 10.1111/brv.12759 34101323

[B33] Chen H. Wang X. Wang Q. (2020). Microalgal biofuels in China: The past, progress and prospects. GCB Bioenergy 00, 1044–1065. 10.1111/gcbb.12741

[B34] Chen L. Liu T. Zhang W. Chen X. Wang J. (2012). Biodiesel production from algae oil high in free fatty acids by two-step catalytic conversion. Bioresour. Technol. 111, 208–214. 10.1016/j.biortech.2012.02.033 22401712

[B35] Chen W. Li T. Ren Y. Wang J. Chen H. Wang Q. (2023). Biological hydrogen with industrial potential: Improvement and prospection in biohydrogen production. J. Clean. Prod. 387, 135777. 10.1016/j.jclepro.2022.135777

[B36] Chen W. Liu Y. Song L. Sommerfeld M. Hu Q. (2020). Automated accelerated solvent extraction method for total lipid analysis of microalgae. Algal Res. 51, 102080. 10.1016/j.algal.2020.102080

[B37] Chen W. Wang J. Ren Y. Chen H. He C. Wang Q. (2021). Optimized production and enrichment of α-linolenic acid by *Scenedesmus* sp. HSJ296. Algal Research-Biomass Biofuels Bioprod. 60, 102505. 10.1016/j.algal.2021.102505

[B38] Chen W. Zhang S. Rong J. Li X. Chen H. He C. (2016). Effective biological DeNOx of industrial flue gas by the mixotrophic cultivation of an oil-producing green alga *Chlorella* sp. C2. Environ. Sci. Technol. 50, 1620–1627. 10.1021/acs.est.5b04696 26751001

[B39] Cheng T. Yang G. Bi Y. (2017). Enrichment of α-linoneic acid by tataric acid-assisted urea fractionation in methanol. J. Henan Univ. Technol. 38, 12–17.

[B40] Chi G. X. Xu Y. Y. Cao X. Y. Li Z. P. Cao M. F. Chisti Y. (2022). Production of polyunsaturated fatty acids by *Schizochytrium (Aurantiochytrium*) spp. Biotechnol. Adv. 55, 107897. 10.1016/j.biotechadv.2021.107897 34974158

[B41] Chiriboga O. Rorrer G. L. (2019). Phosphate addition strategies for enhancing the co-production of lipid and chitin nanofibers during fed-batch cultivation of the diatom *Cyclotella* sp. Algal Research-Biomass Biofuels Bioprod. 38, 101403. 10.1016/j.algal.2018.101403

[B42] Collins A. M. Jones H. D. T. Han D. Hu Q. Beechem T. E. Timlin J. A. (2011). Carotenoid distribution in living cells of *Haematococcus pluvialis* (chlorophyceae). PLoS ONE 6, e24302. 10.1371/journal.pone.0024302 21915307 PMC3167842

[B43] Cooney M. Young G. Nagle N. (2009). Extraction of bio‐oils from microalgae. Sep. Purif. Rev. 38, 291–325. 10.1080/15422110903327919

[B44] Copolovici D. Bungau S. Boscencu R. Tit D. M. Copolovici L. (2017). The fatty acids composition and antioxidant activity of walnut cold press oil. Rev. Chim. 68, 507–509. 10.37358/rc.17.3.5489

[B45] Correia N. Pereira H. Silva J. T. Santos T. Soares M. Sousa C. B. (2020). Isolation, identification and biotechnological applications of a novel, robust, free-living Chlorococcum (*oophila*) amblystomatis strain isolated from a local pond. Appl. Sci. 10, 3040. 10.3390/app10093040

[B46] Corsetto P. A. Montorfano G. Zava S. Jovenitti I. E. Cremona A. Berra B. (2011). Effects of n-3 PUFAs on breast cancer cells through their incorporation in plasma membrane. Lipids Health Dis. 10, 73. 10.1186/1476-511X-10-73 21569413 PMC3127786

[B47] Crexi V. T. Monte M. L. Monte M. L. Pinto L. A. A. (2012). Polyunsaturated fatty acid concentrates of carp oil: Chemical hydrolysis and urea complexation. J. Am. Oil Chemists' Soc. 89, 329–334. 10.1007/s11746-011-1899-4

[B48] Cui Y. Thomas-Hall S. R. Chua E. T. Schenk P. M. (2021). Development of high-level omega-3 eicosapentaenoic acid (EPA) production from *Phaeodactylum tricornutum* . J. Phycol. 57, 258–268. 10.1111/jpy.13082 33025589

[B49] de Souza A. S. Fernandes F. S. Tavares do Carmo M. d. G. (2011). Effects of maternal malnutrition and postnatal nutritional rehabilitation on brain fatty acids, learning, and memory. Nutr. Rev. 69, 132–144. 10.1111/j.1753-4887.2011.00374.x 21348877

[B50] Degraeve-Guilbault C. Pankasem N. Gueirrero M. Lemoigne C. Domergue F. Kotajima T. (2021). Temperature acclimation of the picoalga *Ostreococcus tauri* triggers early fatty-acid variations and involves a plastidial omega 3-desaturase. Front. Plant Sci. 12, 639330. 10.3389/fpls.2021.639330 33815446 PMC8018280

[B51] Dejoye C. Vian M. A. Lumia G. Bouscarle C. Charton F. Chemat F. (2011). Combined extraction processes of lipid from Chlorella vulgaris microalgae: Microwave prior to supercritical carbon dioxide extraction. Int. J. Mol. Sci. 12, 9332–9341. 10.3390/ijms12129332 22272135 PMC3257132

[B52] Derwenskus F. Schäfer B. Müller J. Frick K. Gille A. Briviba K. (2020). Coproduction of EPA and fucoxanthin with P. Tricornutum – a promising approach for up- and downstream processing. Chem. Ing. Tech. 92, 1780–1789. 10.1002/cite.202000046

[B53] Devassy J. G. Leng S. Gabbs M. Monirujjaman M. Aukema H. M. (2016). Omega-3 polyunsaturated fatty acids and oxylipins in neuroinflammation and management of alzheimer disease. Adv. Nutr. 7, 905–916. 10.3945/an.116.012187 27633106 PMC5015035

[B54] Ding D. Shen Z. Ma T. Sun Y. Shi M. Lu T. (2022). Competition of dual roles of ionic liquids during *in situ* transesterification of wet algae. ACS Sustain. Chem. Eng. 10, 13692–13701. 10.1021/acssuschemeng.2c03641

[B55] Djuricic I. Calder P. C. (2021). Beneficial outcomes of omega-6 and omega-3 polyunsaturated fatty acids on human health: An update for 2021. Nutrients 13, 2421. 10.3390/nu13072421 34371930 PMC8308533

[B56] Domart C. Miyauchi D. Sumerwell W. (1955). The fractionation of marine-oil fatty acids with urea. J. Am. Oil Chem. Soc. 32, 481–483. 10.1007/bf02639943

[B57] Dovale-Rosabal G. Rodríguez A. Contreras E. Ortiz-Viedma J. Muñoz M. Trigo M. (2019). Concentration of EPA and DHA from refined salmon oil by optimizing the urea–fatty acid adduction reaction conditions using response surface methodology. Molecules 24, 1642. 10.3390/molecules24091642 31027319 PMC6539647

[B58] Ebm N. Guo F. Brett M. T. Bunn S. E. Kainz M. J. (2021). Polyunsaturated fatty acids in fish tissues more closely resemble algal than terrestrial diet sources. Hydrobiologia 848, 371–383. 10.1007/s10750-020-04445-1 33343020 PMC7738338

[B59] Eleršek T. Flisar K. Likozar B. Klemenčič M. Golob J. Kotnik T. (2020). Electroporation as a solvent-free green technique for non-destructive extraction of proteins and lipids from Chlorella vulgaris. Front. Bioeng. Biotechnol. 8, 443. 10.3389/fbioe.2020.00443 32478057 PMC7237570

[B60] Ellison C. R. Overa S. Boldor D. (2019). Central composite design parameterization of microalgae/cyanobacteria co-culture pretreatment for enhanced lipid extraction using an external clamp-on ultrasonic transducer. Ultrason. Sonochemistry 51, 496–503. 10.1016/j.ultsonch.2018.05.006 29793838

[B61] Eloussaief M. Chakroun S. Kallel N. Benzina M. (2020). Efficiency of clay materials collected from Ain Jeloula (Central Tunisia) in sunflower oil decolorization. Euro-Mediterranean J. Environ. Integration 5, 33. 10.1007/s41207-020-00171-1

[B62] Faraji Sarabmirza R. Joolaei Ahranjani P. Rashidi L. Mousavi M. Khodaiyan F. Rashidi Nodeh H. (2023). An investigation on conjugated linoleic acid content, fatty acid composition, and physicochemical characteristics of Iranian Kurdish butter oil. Food Sci. Nutr. 11, 1051–1058. 10.1002/fsn3.3142 36789035 PMC9922134

[B63] Farmani J. Hamedi M. Safari M. Madadlou A. (2007). Trans-free Iranian vanaspati through enzymatic and chemical transesterification of triple blends of fully hydrogenated soybean, rapeseed and sunflower oils. Food Chem. 102, 827–833. 10.1016/j.foodchem.2006.06.015

[B64] Felix C. Ubando A. Madrazo C. Gue I. H. Sutanto S. Tran-Nguyen P. L. (2019). Non-catalytic *in-situ* (trans) esterification of lipids in wet microalgae *Chlorella vulgaris* under subcritical conditions for the synthesis of fatty acid methyl esters. Appl. Energy 248, 526–537. 10.1016/j.apenergy.2019.04.149

[B65] Ferreira M. Salgado J. M. Fernandes H. Peres H. Belo I. (2022). Potential of red, green and Brown seaweeds as substrates for solid state fermentation to increase their nutritional value and to produce enzymes. Foods 11, 3864. 10.3390/foods11233864 36496673 PMC9741140

[B66] Fonteh A. N. Cipolla M. Chiang A. J. Edminster S. P. Arakaki X. Harrington M. G. (2020). Polyunsaturated fatty acid composition of cerebrospinal fluid fractions shows their contribution to cognitive resilience of a pre-symptomatic Alzheimer’s disease cohort. Front. Physiology 11, 83. 10.3389/fphys.2020.00083 PMC7034243 32116789

[B67] Gao F. Z. Cabanelas I. T. D. Wijffels R. H. Barbosa M. J. (2022). Fucoxanthin and docosahexaenoic acid production by cold-adapted *Tisochrysis lutea* . New Biotechnol. 66, 16–24. 10.1016/j.nbt.2021.08.005 34500104

[B68] Ge F. Song K. Yang Z. Li J. Yan F. Zhang M. (2023). Enhancing docosahexaenoic acid production of *Isochrysis galbana* from starch-rich food processing byproducts. Fermentation 9, 158. 10.3390/fermentation9020158

[B69] Golanski J. Szymanska P. Rozalski M. (2021). Effects of omega-3 polyunsaturated fatty acids and their metabolites on haemostasis—current perspectives in cardiovascular disease. Int. J. Mol. Sci. 22, 2394. 10.3390/ijms22052394 33673634 PMC7957531

[B70] González-Fernández M. J. Fabrikov D. Lyashenko S. Ferrón-Carrillo F. Guil-Guerrero J. L. (2020). Highly concentrated very long-chain PUFA obtainment by Urea complexation methodology. Environ. Technol. Innovation 18, 100736. 10.1016/j.eti.2020.100736

[B71] Grierson S. Strezov V. Bray S. Mummacari R. Danh L. T. Foster N. (2012). Assessment of bio-oil extraction from Tetraselmis chui microalgae comparing supercritical CO_2_, solvent extraction, and thermal processing. Energy & Fuels 26, 248–255. 10.1021/ef2011222

[B72] Gu W. Kavanagh J. M. McClure D. D. (2021). Photoautotrophic production of eicosapentaenoic acid. Crit. Rev. Biotechnol. 41, 731–748. 10.1080/07388551.2021.1888065 33784913

[B73] Guesnet P. Alessandri J.-M. (2011). Docosahexaenoic acid (DHA) and the developing central nervous system (CNS) – implications for dietary recommendations. Biochimie 93, 7–12. 10.1016/j.biochi.2010.05.005 20478353

[B74] Gunstone F. (2011). Vegetable oils in food technology: Composition, properties and uses. New York, United States: John Wiley & Sons.

[B75] Hac İsa M. Metin C. Ercan E. Alparslan Y. (2022). Effect of different cell disruption methods on lipid yield of Schizochytrium sp. J. Am. Oil Chemists' Soc. 99, 129–139. 10.1002/aocs.12551

[B76] Halim R. Harun R. Danquah M. K. Webley P. A. (2012). Microalgal cell disruption for biofuel development. Appl. Energy 91, 116–121. 10.1016/j.apenergy.2011.08.048

[B77] Hamilton H. A. Newton R. Auchterlonie N. A. Müller D. B. (2020). Systems approach to quantify the global omega-3 fatty acid cycle. Nat. Food 1, 59–62. 10.1038/s43016-019-0006-0

[B78] Han D. X. Li Y. T. Hu Q. (2013). Astaxanthin in microalgae: Pathways, functions and biotechnological implications. Algae 28, 131–147. 10.4490/algae.2013.28.2.131

[B79] Han X. Xu P. Meng X. Liang Z. Zhang Z. Yang Z. (2004). Preparation of high-purity linolenic acid from oil of *Lithospermum erythrorhizon* by urea inclusion and column chromatography. J. Chin. Pharm. Sci. 13, 53–57.

[B80] Hashimoto M. Tanabe Y. Hossain S. Matsuzaki K. Ohno M. Kato S. (2020). Intake of alpha-linolenic acid-rich *perilla frutescens* leaf powder decreases home blood pressure and serum oxidized low-density lipoprotein in Japanese adults. Molecules 25, 2099. 10.3390/molecules25092099 32365849 PMC7248687

[B81] Hatlen B. Larsson T. Østbye T.-K. Romarheim O. H. Rubio L. M. Ruyter B. (2022). Improved fillet quality in harvest-size Atlantic salmon fed high n-3 canola oil as a DHA-source. Aquaculture 560, 738555. 10.1016/j.aquaculture.2022.738555

[B82] He J. Hong B. Lu R. Zhang R. Fang H. Huang W. (2020). Separation of saturated fatty acids from docosahexaenoic acid-rich algal oil by enzymatic ethanolysis in tandem with molecular distillation. Food Sci. Nutr. 8, 2234–2241. 10.1002/fsn3.1462 32405380 PMC7215222

[B83] Hidalgo P. Toro C. Ciudad G. Navia R. (2013). Advances in direct transesterification of microalgal biomass for biodiesel production. Rev. Environ. Sci. Bio/Technology 12, 179–199. 10.1007/s11157-013-9308-0

[B84] Hollingsworth M. D. Brown M. E. Hillier A. C. Santarsiero B. D. Chaney J. D. (1996). Superstructure control in the crystal growth and ordering of urea inclusion compounds. Science 273, 1355–1359. 10.1126/science.273.5280.1355

[B85] Hosseinizand H. Sokhansanj S. Lim C. J. (2018). Studying the drying mechanism of microalgae *Chlorella vulgaris* and the optimum drying temperature to preserve quality characteristics. Dry. Technol. 36, 1049–1060. 10.1080/07373937.2017.1369986

[B86] Hoy S. M. Keating G. M. (2009). Omega-3 ethylester concentrate. Drugs 69, 1077–1105. 10.2165/00003495-200969080-00008 19496632

[B87] Huang P. W. Wang L. R. Geng S. S. Ye C. Sun X. M. Huang H. (2021). Strategies for enhancing terpenoids accumulation in microalgae. Appl. Microbiol. Biotechnol. 105, 4919–4930. 10.1007/s00253-021-11368-x 34125275

[B88] Islam M. S. Christopher L. P. Alam M. N. (2020). Separation and purification of ω-6 linoleic acid from crude tall oil. Separations 7, 9. 10.3390/separations7010009

[B89] Jacob J. P. Mathew S. (2017). Effect of lipases from *Candida cylinderacea* on enrichment of PUFA in marine microalgae. J. Food Process. Preserv. 41, e12928. 10.1111/jfpp.12928

[B90] James M. J. Gibson R. A. Cleland L. G. (2000). Dietary polyunsaturated fatty acids and inflammatory mediator production. Am. J. Clin. Nutr. 71, 343S–348S. 10.1093/ajcn/71.1.343s 10617994

[B91] Jeffrey B. G. Mitchell D. C. Gibson R. A. Neuringer M. (2002a). n-3 fatty acid deficiency alters recovery of the rod photoresponse in rhesus monkeys. Investigative Ophthalmol. Vis. Sci. 43, 2806–2814. 10.1007/s00417-002-0511-X 12147619

[B92] Jeffrey B. G. Mitchell D. C. Hibbeln J. R. Gibson R. A. Lee Chedester A. Salem N. (2002b). Visual acuity and retinal function in infant monkeys fed long-chain PUFA. Lipids 37, 839–848. 10.1007/s11745-002-0969-0 12458618

[B93] Jensen G. Ginsberg D. I. Drapeau C. (2001). Blue-green algae as an immuno-enhancer and biomodulator. J. Am. Nutraceutical Assoc. 3, 24–30.

[B94] Jiménez Callejón M. J. Robles Medina A. Macías Sánchez M. D. González Moreno P. A. Navarro López E. Esteban Cerdán L. (2022). Supercritical fluid extraction and pressurized liquid extraction processes applied to eicosapentaenoic acid-rich polar lipid recovery from the microalga Nannochloropsis sp. Algal Res. 61, 102586. 10.1016/j.algal.2021.102586

[B95] Jin H. Chuai W. Li K. Hou G. Wu M. Chen J. (2021). Ultrahigh-cell-density heterotrophic cultivation of the unicellular green alga Chlorella sorokiniana for biomass production. Biotechnol. Bioeng. 118, 4138–4151. 10.1002/bit.27890 34264522

[B96] Jin H. Zhang H. Zhou Z. Li K. Hou G. Xu Q. (2020). Ultrahigh-cell-density heterotrophic cultivation of the unicellular green microalga Scenedesmus acuminatus and application of the cells to photoautotrophic culture enhance biomass and lipid production. Biotechnol. Bioeng. 117, 96–108. 10.1002/bit.27190 31612991 PMC6916281

[B97] Jithu Paul J. (2019). “Bioconcentration of marine algae using lipase enzyme,” in Microalgae. Editor Milada V. (Rijeka: IntechOpen). Ch. 8. 10.5772/intechopen.87026

[B98] Kadalag N. L. Pawar P. R. Prakash G. (2022). Co-cultivation of *Phaeodactylum tricornutum* and *Aurantiochytrium limacinum* for polyunsaturated omega-3 fatty acids production. Bioresour. Technol. 346, 126544. 10.1016/j.biortech.2021.126544 34902489

[B99] Kaveh M. Eftekhar N. Boskabady M. H. (2019). The effect of alpha linolenic acid on tracheal responsiveness, lung inflammation, and immune markers in sensitized rats. Iran. J. basic Med. Sci. 22, 255–261. 10.22038/ijbms.2019.27381.6684 31156785 PMC6528709

[B100] Kazemifard S. Nayebzadeh H. Saghatoleslami N. Safakish E. (2019). Application of magnetic alumina-ferric oxide nanocatalyst supported by KOH for *in-situ* transesterification of microalgae cultivated in wastewater medium. Biomass Bioenergy 129, 105338. 10.1016/j.biombioe.2019.105338

[B101] Khera H. K. Srivastava A. K. (2022). “Chapter 22 - genetic engineering of algae,” in An integration of phycoremediation processes in wastewater treatment. Editors Shah M. Rodriguez-Couto S. De La Cruz C. B. V. Biswas J. (Amsterdam, Netherlands: Elsevier), 487–502. 10.1016/B978-0-12-823499-0.00018-3

[B102] Kim B. Im H. Lee J. W. (2015). *In situ* transesterification of highly wet microalgae using hydrochloric acid. Bioresour. Technol. 185, 421–425. 10.1016/j.biortech.2015.02.092 25769690

[B103] Kim E. S. McCormack P. L. (2014). Icosapent ethyl: A review of its use in severe hypertriglyceridemia. Am. J. Cardiovasc. Drugs 14, 471–478. 10.1007/s40256-014-0099-7 25428605

[B104] Knothe G. Dunn R. O. (2009). A comprehensive evaluation of the melting points of fatty acids and esters determined by differential scanning calorimetry. J. Am. Oil Chemists' Soc. 86, 843–856. 10.1007/s11746-009-1423-2

[B105] Korbecki J. Bobiński R. Dutka M. (2019). Self-regulation of the inflammatory response by peroxisome proliferator-activated receptors. Inflamm. Res. 68, 443–458. 10.1007/s00011-019-01231-1 30927048 PMC6517359

[B106] Kreps F. Vrbiková L. Schmidt Š. (2014). Influence of industrial physical refining on tocopherol, chlorophyll and beta‐carotene content in sunflower and rapeseed oil. Eur. J. Lipid Sci. Technol. 116, 1572–1582. 10.1002/ejlt.201300460

[B107] Kris-Etherton P. M. Harris W. S. Appel L. J. (2002). Fish consumption, fish oil, omega-3 fatty acids, and cardiovascular disease. Circulation 106, 2747–2757. 10.1161/01.CIR.0000038493.65177.94 12438303

[B108] Kugler A. Zorin B. Didi-Cohen S. Sibiryak M. Gorelova O. Ismagulova T. (2019). Long-chain polyunsaturated fatty acids in the green microalga *Lobosphaera incisa* contribute to tolerance to abiotic stresses. Plant Cell Physiology 60, 1205–1223. 10.1093/pcp/pcz013 30668793

[B109] Kumar N. Varun Chauhan S. R. (2013). Performance and emission characteristics of biodiesel from different origins: A review. Renew. Sustain. Energy Rev. 21, 633–658. 10.1016/j.rser.2013.01.006

[B110] Lee J.-Y. Yoo C. Jun S.-Y. Ahn C.-Y. Oh H.-M. (2010). Comparison of several methods for effective lipid extraction from microalgae. Bioresour. Technol. 101, S75–S77. 10.1016/j.biortech.2009.03.058 19386486

[B111] Lee R. E. (2018). Phycology. 5 edn. Cambridge: Cambridge University Press.

[B112] Lehninger A. L. (1982). Principles of biochemistry. New York: Worth Publishers Inc.

[B113] Li S. Hu T. Xu Y. Wang J. Chu R. Yin Z. (2020). A review on flocculation as an efficient method to harvest energy microalgae: Mechanisms, performances, influencing factors and perspectives. Renew. Sustain. Energy Rev. 131, 110005. 10.1016/j.rser.2020.110005

[B114] Li X. Liu J. Chen G. Zhang J. Wang C. Liu B. (2019). Extraction and purification of eicosapentaenoic acid and docosahexaenoic acid from microalgae: A critical review. Algal Res. 43, 101619. 10.1016/j.algal.2019.101619

[B115] Li Y Y. Sun H. Wu T. Fu Y. He Y. Mao X. (2019). Storage carbon metabolism of Isochrysis zhangjiangensis under different light intensities and its application for co-production of fucoxanthin and stearidonic acid. Bioresour. Technol. 282, 94–102. 10.1016/j.biortech.2019.02.127 30852337

[B116] Liu C.-Z. Zheng S. Xu L. Wang F. Guo C. (2013). Algal oil extraction from wet biomass of Botryococcus braunii by 1,2-dimethoxyethane. Appl. Energy 102, 971–974. 10.1016/j.apenergy.2012.08.016

[B117] Liu G. R. Chen G. Y. (2012). Study on the decolorization of biodiesel from waste cooking oil. Adv. Mater. Res. 347-353, 3781–3787. 10.4028/www.scientific.net/amr.347-353.3781.347-353.3781

[B118] Liu J. Mao X. Zhou W. Guarnieri M. T. (2016). Simultaneous production of triacylglycerol and high-value carotenoids by the astaxanthin-producing oleaginous green microalga *Chlorella zofingiensis* . Bioresour. Technol. 214, 319–327. 10.1016/j.biortech.2016.04.112 27152772

[B119] Liu N. Li D. Wang W. Hollmann F. Xu L. Ma Y. (2018). Production and immobilization of lipase PCL and its application in synthesis of α-linolenic acid-rich diacylglycerol. J. Food Biochem. 42, e12574. 10.1111/jfbc.12574

[B120] Liu Y. Zheng L. (2014). The comparison of the quality and comprehensive decolorization effect of crude corn germ oil obtained respectively by pre-pressing and leaching process. Cereal & Food Industry 21, 6–13.

[B121] Ljubic A. Thulesen E. T. Jacobsen C. Jakobsen J. (2021). UVB exposure stimulates production of vitamin D-3 in selected microalgae. Algal Research-Biomass Biofuels Bioprod. 59, 102472. 10.1016/j.algal.2021.102472

[B122] Lourenço-Lopes C. Garcia-Oliveira P. Carpena M. Fraga-Corral M. Jimenez-Lopez C. Pereira A. G. (2020). Scientific approaches on extraction, purification and stability for the commercialization of fucoxanthin recovered from Brown algae. Foods 9, 1113. 10.3390/foods9081113 32823574 PMC7465967

[B123] Lu X. Liu B. He Y. Guo B. Sun H. Chen F. (2019). Novel insights into mixotrophic cultivation of *Nitzschia laevis* for co-production of fucoxanthin and eicosapentaenoic acid. Bioresour. Technol. 294, 122145. 10.1016/j.biortech.2019.122145 31539854

[B124] Ma R. You Y. Liu X. Ho S.-H. Xie Y. Chen J. (2023). Highly efficient co-production of fucoxanthin and eicosapentaenoic acid by heterotrophic cultivation of a newly isolated microalga Nitzschia sp. FZU62. Algal Res. 71, 103046. 10.1016/j.algal.2023.103046

[B125] Ma X.-N. Chen T.-P. Yang B. Liu J. Chen F. (2016). Lipid production from *Nannochloropsis* . Mar. Drugs 14, 61. 10.3390/md14040061 27023568 PMC4849066

[B126] Madany M. A. Abdel-Kareem M. S. Al-Oufy A. K. Haroun M. Sheweita S. A. (2021). The biopolymer ulvan from Ulva fasciata: Extraction towards nanofibers fabrication. Int. J. Biol. Macromol. 177, 401–412. 10.1016/j.ijbiomac.2021.02.047 33577821

[B127] Magpusao J. Oey I. Kebede B. (2022). Changes in volatile and fatty acid compositions of selected microalgal suspensions with high pressure homogenization. Algal Res. 67, 102867. 10.1016/j.algal.2022.102867

[B128] Mahsa K. Akram E. Ali N. Mohammad Hossein B. (2017). The extract of *Portulaca oleracea* and its constituent, alpha linolenic acid affects serum oxidant levels and inflammatory cells in sensitized rats. Iran. J. Allergy, Asthma Immunol. 16, 256–270. 28732439

[B129] Mao X. Wu T. Sun D. Zhang Z. Chen F. (2018). Differential responses of the green microalga *Chlorella zofingiensis* to the starvation of various nutrients for oil and astaxanthin production. Bioresour. Technol. 249, 791–798. 10.1016/j.biortech.2017.10.090 29136934

[B130] Marella T. K. Bhattacharjya R. Tiwari A. (2021). Impact of organic carbon acquisition on growth and functional biomolecule production in diatoms. Microb. Cell Factories 20, 135. 10.1186/s12934-021-01627-x PMC8281487 34266439

[B131] Matsui H. Sugihara S. Wada M. Ozaki T. Saitoh T. Kotani T. (2022). Application of genetic disruption of a *Nannochloropsis oceanica* cell wall synthesizing gene to n-3 HUFA enrichment of *Brachionus plicatilis* . Aquaculture 552, 738022. 10.1016/j.aquaculture.2022.738022

[B132] Mejía-Zepeda R. Pérez-Hernández I. H. (2020). Effect of alpha linolenic acid on membrane fluidity and respiration of liver mitochondria in normoglycemic and diabetic Wistar rats. J. Bioenergetics Biomembr. 52, 421–430. 10.1007/s10863-020-09859-z 33156468

[B133] Mendes M. C. Navalho S. Ferreira A. Paulino C. Figueiredo D. Silva D. (2022). Algae as food in europe: An overview of species diversity and their application. Foods 11, 1871. 10.3390/foods11131871 35804686 PMC9265617

[B134] Menegol T. Romero-Villegas G. I. López-Rodríguez M. Navarro-López E. López-Rosales L. Chisti Y. (2019). Mixotrophic production of polyunsaturated fatty acids and carotenoids by the microalga Nannochloropsis gaditana. J. Appl. Phycol. 31, 2823–2832. 10.1007/s10811-019-01828-3

[B135] Meng X. Gao H. Kong R. Xu X. (2018). Optimization of heterotrophic growth conditions for a *Scenedesmus* strain with high content of α-linolenic acid. Acta Hydrobiol. Sin. 42, 819–823. 10.7541/2018.100

[B136] Mercer P. Armenta R. E. (2011). Developments in oil extraction from microalgae. Eur. J. Lipid Sci. Technol. 113, 539–547. 10.1002/ejlt.201000455

[B137] Metzner H. (1984). Geoffrey zubay (coordinating author). Reading, Menlo Park, London, Amsterdam, Don Mills, Sydney: Addison-Wesley Publishing Company. 10.1016/0302-4598(84)87027-0 Biochemistry

[B138] Mizukami T. Ikeda K. Shimanaka Y. Korogi K. Zhou C. Takase H. (2018). Reelin deficiency leads to aberrant lipid composition in mouse brain. Biochem. Biophysical Res. Commun. 505, 81–86. 10.1016/j.bbrc.2018.09.089 30241938

[B139] Mohamadzadeh Shirazi H. Karimi-Sabet J. Ghotbi C. (2017). Biodiesel production from *Spirulina* microalgae feedstock using direct transesterification near supercritical methanol condition. Bioresour. Technol. 239, 378–386. 10.1016/j.biortech.2017.04.073 28531863

[B140] Moharana T. R. Rao N. M. (2020). Substrate structure and computation guided engineering of a lipase for omega-3 fatty acid selectivity. PLOS ONE 15, e0231177. 10.1371/journal.pone.0231177 32271820 PMC7145112

[B141] Molino A. Mehariya S. Di Sanzo G. Larocca V. Martino M. Leone G. P. (2020). Recent developments in supercritical fluid extraction of bioactive compounds from microalgae: Role of key parameters, technological achievements and challenges. J. CO_2_ Util. 36, 196–209. 10.1016/j.jcou.2019.11.014

[B142] Moniz P. Silva C. Oliveira A. C. Reis A. Lopes da Silva T. (2021). Raw glycerol based medium for DHA and lipids production, using the marine heterotrophic microalga *Crypthecodinium cohnii* . Processes 9, 2005. 10.3390/pr9112005

[B143] Muhlhausler B. S. Collins C. T. Gould J. F. Best K. P. Leghi G. E. (2018). “Chapter 7 - polyunsaturated fatty acids: Metabolism and nutritional requirements in pregnancy and infancy,” in Polyunsaturated fatty acid metabolism. Editor Burdge G. C. (Urbana: AOCS Press), 111–134. 10.1016/B978-0-12-811230-4.00007-7

[B144] Muramatsu T. Yatsuya H. Toyoshima H. Sasaki S. Li Y. Otsuka R. (2010). Higher dietary intake of alpha-linolenic acid is associated with lower insulin resistance in middle-aged Japanese. Prev. Med. 50, 272–276. 10.1016/j.ypmed.2010.02.014 20211645

[B145] Mwakasege E. Treydte A. Hoeglinger O. Kassim N. Makule E. (2021). Fatty acid contents and stability of oyster nut oil (*Telfairia pedata*) compared to flaxseed and sunflower oil. Int. J. Food Sci. 2021, 1–8. 10.1155/2021/9985910 PMC8601856 34805397

[B146] Nagappan S. Devendran S. Tsai P.-C. Dinakaran S. Dahms H.-U. Ponnusamy V. K. (2019). Passive cell disruption lipid extraction methods of microalgae for biofuel production – a review. Fuel 252, 699–709. 10.1016/j.fuel.2019.04.092

[B147] Nagappan S. Kumar Verma S. (2018). Co-production of biodiesel and alpha-linolenic acid (omega-3 fatty acid) from microalgae,*Desmodesmus sp.*MCC34. Desmodesmus sp. MCC34. Energy Sources, Part A Recovery, Util. Environ. Eff. 40, 2933–2940. 10.1080/15567036.2018.1514434

[B148] Najar-Villarreal F. Boyle E. Danler R. O’quinn T. Houser T. Gonzalez J. (2019). Fatty acid composition, proximate analysis, and consumer sensory evaluation of United States retail grass-fed ground beef. Meat Muscle Biol. 3, 18. 10.22175/mmb2019.06.0018

[B149] Napier J. A. (2002). Plumbing the depths of PUFA biosynthesis: A novel polyketide synthase-like pathway from marine organisms. Trends Plant Sci. 7, 51–54. 10.1016/S1360-1385(01)02191-4 11832269

[B150] Neag E. Stupar Z. Maicaneanu S. A. Roman C. (2023). Advances in biodiesel production from microalgae. Energies 16, 1129. 10.3390/en16031129

[B151] Neuringer M. Connor W. E. Lin D. S. Barstad L. Luck S. (1986). Biochemical and functional effects of prenatal and postnatal omega 3 fatty acid deficiency on retina and brain in rhesus monkeys. Proc. Natl. Acad. Sci. 83, 4021–4025. 10.1073/pnas.83.11.4021 3459166 PMC323657

[B152] Nichols D. S. (2003). Prokaryotes and the input of polyunsaturated fatty acids to the marine food web. FEMS Microbiol. Lett. 219, 1–7. 10.1016/s0378-1097(02)01200-4 12594015

[B153] Olkiewicz M. Caporgno M. P. Font J. Legrand J. Lepine O. Plechkova N. V. (2015). A novel recovery process for lipids from microalgæ for biodiesel production using a hydrated phosphonium ionic liquid. Green Chem. 17, 2813–2824. 10.1039/C4GC02448F

[B154] Othman F. S. Jamaluddin H. Ibrahim Z. Hara H. Yahya N. A. Mohamad K. I. S. E. (2019). Production of a-linolenic acid by an oleaginous green algae *Acutodesmus obliquus* isolated from Malaysia. J. Pure Appl. Microbiol. 13, 1297–1306. 10.22207/jpam.13.3.01

[B155] Özcan M. M. Duman E. Duman S. (2021). Influence of refining stages on the physicochemical properties and phytochemicals of canola oil. J. Food Process. Preserv. 45, e15164. 10.1111/jfpp.15164

[B156] Pan X. Xu L. Zhang Y. Xiao X. Wang X. Liu Y. (2012). Efficient display of active *Geotrichum* sp. lipase on Pichia pastoris cell wall and its application as a whole-cell biocatalyst to enrich EPA and DHA in fish oil. J. Agric. Food Chem. 60, 9673–9679. 10.1021/jf301827y 22934819

[B157] Park J.-Y. Oh Y.-K. Lee J.-S. Lee K. Jeong M.-J. Choi S.-A. (2014). Acid-catalyzed hot-water extraction of lipids from *Chlorella vulgaris* . Bioresour. Technol. 153, 408–412. 10.1016/j.biortech.2013.12.065 24393546

[B158] Park J. Kim B. Chang Y. K. Lee J. W. (2017). Wet *in situ* transesterification of microalgae using ethyl acetate as a co-solvent and reactant. Bioresour. Technol. 230, 8–14. 10.1016/j.biortech.2017.01.027 28142105

[B159] Paschos G. K. Magkos F. Panagiotakos D. B. Votteas V. Zampelas A. (2007). Dietary supplementation with flaxseed oil lowers blood pressure in dyslipidaemic patients. Eur. J. Clin. Nutr. 61, 1201–1206. 10.1038/sj.ejcn.1602631 17268413

[B160] Pawar P. R. Velani S. Kumari S. Lali A. M. Prakash G. (2021). Isolation and optimization of a novel thraustochytrid strain for DHA rich and astaxanthin comprising biomass as aquafeed supplement. 3 Biotech. 11, 71. 10.1007/s13205-020-02616-4 PMC7806681 33489688

[B161] Peltomaa E. Johnson M. D. Taipale S. J. (2018). Marine cryptophytes are great sources of EPA and DHA. Mar. Drugs 16, 3. 10.3390/md16010003 PMC5793051 29278384

[B162] Petursdottir A. L. Farr S. A. Morley J. E. Banks W. A. Skuladottir G. V. (2008). Effect of dietary n-3 polyunsaturated fatty acids on brain lipid fatty acid composition, learning ability, and memory of senescence-accelerated mouse. Journals Gerontology Ser. A 63, 1153–1160. 10.1093/gerona/63.11.1153 19038829

[B163] Praveenkumar R. Lee K. Lee J. Oh Y.-K. (2015). Breaking dormancy: An energy-efficient means of recovering astaxanthin from microalgae. Green Chem. 17, 1226–1234. 10.1039/C4GC01413H

[B164] Quilodrán B. Hinzpeter I. Hormazabal E. Quiroz A. Shene C. (2010). Docosahexaenoic acid (C22:6n−3, DHA) and astaxanthin production by *Thraustochytriidae* sp. AS4-A1 a native strain with high similitude to *Ulkenia* sp.: Evaluation of liquid residues from food industry as nutrient sources. Enzyme Microb. Technol. 47, 24–30. 10.1016/j.enzmictec.2010.04.002

[B165] Rajaram S. (2014). Health benefits of plant-derived α-linolenic acid. Am. J. Clin. Nutr. 100, 443S–448S. 10.3945/ajcn.113.071514 24898228

[B166] Ramluckan K. Moodley K. G. Bux F. (2014). An evaluation of the efficacy of using selected solvents for the extraction of lipids from algal biomass by the soxhlet extraction method. Fuel 116, 103–108. 10.1016/j.fuel.2013.07.118

[B167] Ranjan A. Patil C. Moholkar V. S. (2010). Mechanistic assessment of microalgal lipid extraction. Industrial Eng. Chem. Res. 49, 2979–2985. 10.1021/ie9016557

[B168] Raper N. R. Cronin F. J. Exler J. (1992). Omega-3 fatty acid content of the US food supply. J. Am. Coll. Nutr. 11, 304–308. 10.1080/07315724.1992.10718231 1619182

[B169] Rashid N. Park W. K. Selvaratnam T. (2018). Binary culture of microalgae as an integrated approach for enhanced biomass and metabolites productivity, wastewater treatment, and bioflocculation. Chemosphere 194, 67–75. 10.1016/j.chemosphere.2017.11.108 29197817

[B170] Ratledge C. (2001). Microbial lipids. Biotechnol. Set. 2001, 133–197. 10.1002/9783527620999.ch4g

[B171] Rawat I. Ranjith Kumar R. Mutanda T. Bux F. (2011). Dual role of microalgae: Phycoremediation of domestic wastewater and biomass production for sustainable biofuels production. Appl. Energy 88, 3411–3424. 10.1016/j.apenergy.2010.11.025

[B172] Rezanka T. Vitova M. Kolouchova I. Nedbalova L. Dolezalova J. Palyzova A. (2017). Polydatin and its derivatives inhibit fatty acid desaturases in microorganisms. Eur. J. Lipid Sci. Technol. 119, 1600369. 10.1002/ejlt.201600369

[B173] Rodríguez-España M. Mendoza-Sánchez L. G. Magallón-Servín P. Salgado-Cervantes M. A. Acosta-Osorio A. A. García H. S. (2022). Supercritical fluid extraction of lipids rich in DHA from Schizochytrium sp. J. Supercrit. Fluids 179, 105391. 10.1016/j.supflu.2021.105391

[B174] Rodríguez-Miranda J. Gallegos-Marín I. Hernández-Santos B. Herman-Lara E. Medina-Juárez L. A. Juárez-Barrientos J. M. (2019). Effect of frying and storage on oxidative quality of conjugated linoleic-acid-rich soybean oil produced by photoisomerization using plantain as a model system. J. Sci. Food Agric. 99, 3910–3916. 10.1002/jsfa.9614 30693524

[B175] Ronda S. R. Bokka C. S. Ketineni C. Rijal B. Allu P. R. (2012). Aeration effect on spirulina platensis growth and gamma-linolenic acid production. Braz. J. Microbiol. 43, 12–20. 10.1590/s1517-83822012000100002 24031799 PMC3768970

[B176] Ruiz J. Wijffels R. H. Dominguez M. Barbosa M. J. (2022). Heterotrophic vs autotrophic production of microalgae: Bringing some light into the everlasting cost controversy. Algal Res. 64, 102698. 10.1016/j.algal.2022.102698

[B177] Rupani B. Kodam K. Gadre R. Najafpour G. D. (2012). Lipase-mediated hydrolysis of flax seed oil for selective enrichment of α-linolenic acid. Eur. J. Lipid Sci. 114, 1246–1253. 10.1002/ejlt.201100384

[B178] Safi C. Camy S. Frances C. Varela M. M. Badia E. C. Pontalier P.-Y. (2014). Extraction of lipids and pigments of *Chlorella vulgaris* by supercritical carbon dioxide: Influence of bead milling on extraction performance. J. Appl. Phycol. 26, 1711–1718. 10.1007/s10811-013-0212-3

[B179] Salem N. Jr Kim H.-Y. Yergey J. A. (1986). “Chapter 15 - docosahexaenoic acid: Membrane function and metabolism,” in Health effects of polyunsaturated fatty acids in seafoods. Editors Kifer R. R. Martin R. E. (Massachusetts, United Statespp: Academic Press), 263–317. 10.1016/B978-0-12-644360-8.50019-4

[B180] Salminen H. Aulbach S. Leuenberger B. H. Tedeschi C. Weiss J. (2014). Influence of surfactant composition on physical and oxidative stability of Quillaja saponin-stabilized lipid particles with encapsulated ω-3 fish oil. Colloids Surfaces B Biointerfaces 122, 46–55. 10.1016/j.colsurfb.2014.06.045 25016544

[B181] Scanferlato R. Bortolotti M. Sansone A. Chatgilialoglu C. Polito L. De Spirito M. (2019). Hexadecenoic fatty acid positional isomers and de novo PUFA synthesis in colon cancer cells. Int. J. Mol. Sci. 20, 832. 10.3390/ijms20040832 30769921 PMC6412212

[B182] Schmid M. Guihéneuf F. Stengel D. B. (2014). Fatty acid contents and profiles of 16 macroalgae collected from the Irish Coast at two seasons. J. Appl. Phycol. 26, 451–463. 10.1007/s10811-013-0132-2

[B183] Señoráns M. Castejón N. Señoráns F. J. (2020). Advanced extraction of lipids with DHA from *Isochrysis galbana* with enzymatic pre-treatment combined with pressurized liquids and ultrasound assisted extractions. Molecules 25, 3310. 10.3390/molecules25143310 32708275 PMC7397065

[B184] Shen J. Li S. Caydamli Y. Narayanan G. Zhang N. Harrison O. (2018). The role of polymer crystallizability on the formation of polymer-urea-inclusion compounds. Cryst. Growth & Des. 18, 3099–3106. 10.1021/acs.cgd.8b00240

[B185] Shim S. J. Hong M. E. Chang W. S. Sim S. J. (2020). Repeated-batch production of omega-3 enriched biomass of *Chlorella sorokiniana* via calcium-induced homeoviscous adaptation. Bioresour. Technol. 303, 122944. 10.1016/j.biortech.2020.122944 32044645

[B186] Sigurgisladottir S. Lall S. P. Parrish C. C. Ackman R. G. (1992). Cholestane as a digestibility marker in the absorption of polyunsaturated fatty acid ethyl esters in Atlantic salmon. Lipids 27, 418–424. 10.1007/BF02536382 1630276

[B187] Simopoulos A. P. Leaf A. Salem N. Jr (2000). Workshop statement on the essentiality of and recommended dietary intakes for Omega-6 and Omega-3 fatty acids. Prostagl. Leukot. Essent. Fat. Acids 63, 119–121. 10.1054/plef.2000.0176 10991764

[B188] Simopoulos A. P. (1991). Omega-3 fatty acids in health and disease and in growth and development. Am. J. Clin. Nutr. 54, 438–463. 10.1093/ajcn/54.3.438 1908631

[B189] Sivaramakrishnan R. Incharoensakdi A. (2019). Enhancement of lipid extraction for efficient methyl ester production from Chlamydomonas sp. J. Appl. Phycol. 31, 2365–2377. 10.1007/s10811-019-1758-5

[B190] Smink W. Gerrits W. J. J. Gloaguen M. Ruiter A. van Baal J. (2012). Linoleic and α-linolenic acid as precursor and inhibitor for the synthesis of long-chain polyunsaturated fatty acids in liver and brain of growing pigs. Animal 6, 262–270. 10.1017/S1751731111001479 22436184

[B191] Solana M. Rizza C. S. Bertucco A. (2014). Exploiting microalgae as a source of essential fatty acids by supercritical fluid extraction of lipids: Comparison between *Scenedesmus obliquus, Chlorella protothecoides* and *Nannochloropsis salina* . J. Supercrit. Fluids 92, 311–318. 10.1016/j.supflu.2014.06.013

[B192] Soleimanian Y. Sahari M. A. Barzegar M. (2015). Influence of processing parameters on physicochemical properties of fractionated fish oil at low temperature crystallization. Nutr. Food Sci. 45, 2–19. 10.1108/NFS-05-2014-0038

[B193] Spurvey S. A. Shahidi F. (2000). Concentration of gamma linolenic acid (gla) from borage oil by urea complexation: Optimization of reaction conditions. J. Food Lipids 7, 163–174. 10.1111/j.1745-4522.2000.tb00169.x

[B194] Stivala S. Reiner M. F. Lohmann C. Lüscher T. F. Matter C. M. Beer J. H. (2013). Dietary α-linolenic acid increases the platelet count in ApoE^−/−^ mice by reducing clearance. Blood 122, 1026–1033. 10.1182/blood-2013-02-484741 23801636

[B195] Stramarkou M. Oikonomopoulou V. Chalima A. Boukouvalas C. Topakas E. Krokida M. (2021). Optimization of green extractions for the recovery of docosahexaenoic acid (DHA) from *Crypthecodinium cohnii* . Algal Research-Biomass Biofuels Bioprod. 58, 102374. 10.1016/j.algal.2021.102374

[B196] Su H. Wang X. Kim Y. G. Kim S. B. Seo Y.-G. Kim J. S. (2014). Optimization of decoloring conditions of crude fatty acids recovered from crude glycerol by acid-activated clay using response surface method. Korean J. Chem. Eng. 31, 2070–2076. 10.1007/s11814-014-0158-4

[B197] Sun J. Zhou C. Cheng P. Zhu J. Hou Y. Li Y. (2022). A simple and efficient strategy for fucoxanthin extraction from the microalga *Phaeodactylum tricornutum* . Algal Res. 61, 102610. 10.1016/j.algal.2021.102610

[B198] Swanson D. Block R. Mousa S. A. (2012). Omega-3 fatty acids EPA and DHA: Health benefits throughout life. Adv. Nutr. 3, 1–7. 10.3945/an.111.000893 22332096 PMC3262608

[B199] Tachihana S. Nagao N. Katayama T. Hirahara M. Yusoff F. M. Banerjee S. (2020). High productivity of eicosapentaenoic acid and fucoxanthin by a marine diatom Chaetoceros gracilis in a semi-continuous culture. Front. Bioeng. Biotechnol. 8, 602721. 10.3389/fbioe.2020.602721 33363132 PMC7759640

[B200] Teo C. L. Idris A. (2014). Enhancing the various solvent extraction method via microwave irradiation for extraction of lipids from marine microalgae in biodiesel production. Bioresour. Technol. 171, 477–481. 10.1016/j.biortech.2014.08.024 25201293

[B201] Uemura H. (2012). Synthesis and production of unsaturated and polyunsaturated fatty acids in yeast: Current state and perspectives. Appl. Microbiol. Biotechnol. 95, 1–12. 10.1007/s00253-012-4105-1 22562166

[B202] Umamaheswari J. Kavitha M. S. Shanthakumar S. (2020). Outdoor cultivation of Chlorella pyrenoidosa in paddy-soaked wastewater and a feasibility study on biodiesel production from wet algal biomass through *in-situ* transesterification. Biomass Bioenergy 143, 105853. 10.1016/j.biombioe.2020.105853

[B203] Umezawa M. Kogishi K. Tojo H. Yoshimura S. Seriu N. Ohta A. (1999). High-linoleate and high-α-linolenate diets affect learning ability and natural behavior in SAMR1 mice. J. Nutr. 129, 431–437. 10.1093/jn/129.2.431 10024623

[B204] Valizadeh Derakhshan M. Nasernejad B. Dadvar M. Hamidi M. (2014). Pretreatment and kinetics of oil extraction from algae for biodiesel production. Asia-Pacific J. Chem. Eng. 9, 629–637. 10.1002/apj.1790

[B205] Valverde L. M. Moreno P. A. G. Callejón M. J. J. Cerdán L. E. Medina A. R. (2013). Concentration of eicosapentaenoic acid (EPA) by selective alcoholysis catalyzed by lipases. Eur. J. Lipid Sci. Technol. 115, 990–1004. 10.1002/ejlt.201300005

[B206] Valverde L. M. Moreno P. A. G. Cerdán L. E. López E. N. López B. C. Medina A. R. (2014). Concentration of docosahexaenoic and eicosapentaenoic acids by enzymatic alcoholysis with different acyl-acceptors. Biochem. Eng. J. 91, 163–173. 10.1016/j.bej.2014.08.010

[B207] Varin A. Thomas C. Ishibashi M. Ménégaut L. Gautier T. Trousson A. (2015). Liver X receptor activation promotes polyunsaturated fatty acid synthesis in macrophages. Arteriosclerosis, Thrombosis, Vasc. Biol. 35, 1357–1365. 10.1161/ATVBAHA.115.305539 25838428

[B208] Vidyashankar S. VenuGopal K. S. Swarnalatha G. V. Kavitha M. D. Chauhan V. S. Ravi R. (2015). Characterization of fatty acids and hydrocarbons of chlorophycean microalgae towards their use as biofuel source. Biomass Bioenergy 77, 75–91. 10.1016/j.biombioe.2015.03.001

[B209] Wang J. Zhang J.-L. Wu F.-A. (2013). Enrichment process for α-linolenic acid from silkworm pupae oil. Eur. J. Lipid Sci. Technol. 115, 791–799. 10.1002/ejlt.201200324

[B210] Wang J. Zhou W. Chen H. Zhan J. He C. Wang Q. (2019). Ammonium nitrogen tolerant *Chlorella* strain screening and its damaging effects on photosynthesis. Front. Microbiol. 9, 3250. 10.3389/fmicb.2018.03250 30666245 PMC6330332

[B211] Wang M. Ma L. Yang Y. Xiao Z. Wan J. (2019). n-3 Polyunsaturated fatty acids for the management of alcoholic liver disease: A critical review. Crit. Rev. Food Sci. Nutr. 59, S116–S129. 10.1080/10408398.2018.1544542 30580553

[B212] Wang S. Yerkebulan M. Abomohra A. E.-F. El-Khodary S. Wang Q. (2019). Microalgae harvest influences the energy recovery: A case study on chemical flocculation of *Scenedesmus obliquus* for biodiesel and crude bio-oil production. Bioresour. Technol. 286, 121371. 10.1016/j.biortech.2019.121371 31030071

[B213] Wang X X. Fosse H. K. Li K. Chauton M. S. Vadstein O. Reitan K. I. (2019). Influence of nitrogen limitation on lipid accumulation and EPA and DHA content in four marine microalgae for possible use in aquafeed. Front. Mar. Sci. 6. 10.3389/fmars.2019.00095

[B214] Ward O. P. Singh A. (2005). Omega-3/6 fatty acids: Alternative sources of production. Process Biochem. 40, 3627–3652. 10.1016/j.procbio.2005.02.020

[B215] Wayan Suriani N. Komansilan A. (2019). Enrichment of omega-3 fatty acids, waste oil by-products canning tuna (thunnus sp.) with urea crystallization. J. Phys. Conf. Ser. 1317, 012056. 10.1088/1742-6596/1317/1/012056

[B216] Wei B. Wang S. (2020). Separation of eicosapentaenoic acid and docosahexaenoic acid by three-zone simulated moving bed chromatography. J. Chromatogr. A 1625, 461326. 10.1016/j.chroma.2020.461326 32709355

[B217] Winnik S. Lohmann C. Richter E. K. Schäfer N. Song W.-L. Leiber F. (2011). Dietary α-linolenic acid diminishes experimental atherogenesis and restricts T cell-driven inflammation. Eur. Heart J. 32, 2573–2584. 10.1093/eurheartj/ehq501 21285075 PMC3195262

[B218] Wu C. e. Xu K. Li Y. Luo G. Li T. (2005). Enrichment of α-linolenic acid from seed oil of kiwi fruit based on urea adduction fractionation method. Trans. Chin. Soc. Agric. Mach. 36, 57–61.

[B219] Xia Y. Zhang Y.-T. Sun J.-Y. Huang H. Zhao Q. Ren L.-J. (2020). Strategies for enhancing eicosapentaenoic acid production: From fermentation to metabolic engineering. Algal Res. 51, 102038. 10.1016/j.algal.2020.102038

[B220] Xie D. Jackson E. N. Zhu Q. (2015). Sustainable source of omega-3 eicosapentaenoic acid from metabolically engineered yarrowia lipolytica: From fundamental research to commercial production. Appl. Microbiol. Biotechnol. 99, 1599–1610. 10.1007/s00253-014-6318-y 25567511 PMC4322222

[B221] Xue Z. Sharpe P. L. Hong S.-P. Yadav N. S. Xie D. Short D. R. (2013). Production of omega-3 eicosapentaenoic acid by metabolic engineering of Yarrowia lipolytica. Nat. Biotechnol. 31, 734–740. 10.1038/nbt.2622 23873085

[B222] Yamashita T. Oda E. Sano T. Yamashita T. Ijiru Y. Giddings J. C. (2005). Varying the ratio of dietary n−6/n−3 polyunsaturated fatty acid alters the tendency to thrombosis and progress of atherosclerosis in apoE−/− LDLR−/− double knockout mouse. Thrombosis Res. 116, 393–401. 10.1016/j.thromres.2005.01.011 16122552

[B223] Yang B. Ren X.-L. Fu Y.-Q. Gao J.-L. Li D. (2014). Ratio of n-3/n-6 PUFAs and risk of breast cancer: A meta-analysis of 274135 adult females from 11 independent prospective studies. BMC Cancer 14, 105. 10.1186/1471-2407-14-105 24548731 PMC4016587

[B224] Yang F F. Yuan W. S. Q. Ma Y. H. Balamurugan S. Li H. Y. Fu S. L. (2019). Harnessing the lipogenic potential of delta 6-desaturase for simultaneous hyperaccumulation of lipids and polyunsaturated fatty acids in *Nannochloropsis oceanica* . Front. Mar. Sci. 6. 10.3389/fmars.2019.00682

[B225] Yang J. Wen C. Duan Y. Deng Q. Peng D. Zhang H. (2021). The composition, extraction, analysis, bioactivities, bioavailability and applications in food system of flaxseed (*Linum usitatissimum* L.) oil: A review. Trends Food Sci. Technol. 118, 252–260. 10.1016/j.tifs.2021.09.025

[B226] Yang L.-Y. Kuksis A. Myher J. J. (1989). Lumenal hydrolysis of menhaden and rapeseed oils and their fatty acid methyl and ethyl esters in the rat. Biochem. Cell Biol. 67, 192–204. 10.1139/o89-030 2775528

[B227] Yang M. Sun H. Peng T. Li B. Xie Y. (2019). Structural alteration of montmorillonite by acid activation and its effect on the decolorization of rapeseed oil. J. Minerals 71, 3667–3672. 10.1007/s11837-019-03665-8

[B228] Yang X. Li Y. Li Y. Ye D. Yuan L. Sun Y. (2019). Solid matrix-supported supercritical CO_2_ enhances extraction of γ-linolenic acid from the cyanobacterium arthrospira (*spirulina*) platensis and bioactivity evaluation of the molecule in zebrafish. Mar. Drugs 17, 203. 10.3390/md17040203 30935028 PMC6520994

[B229] Yew G. Y. Tan X. Chew K. W. Chang J.-S. Tao Y. Jiang N. (2021). Thermal-Fenton mechanism with sonoprocessing for rapid non-catalytic transesterification of microalgal to biofuel production. Chem. Eng. J. 408, 127264. 10.1016/j.cej.2020.127264

[B230] Yin F.-W. Zhan C.-T. Huang J. Sun X.-L. Yin L.-F. Zheng W.-L. (2023). Efficient Co-production of docosahexaenoic acid oil and carotenoids in Aurantiochytrium sp. using a light intensity gradient strategy. Appl. Biochem. Biotechnol. 195, 623–638. 10.1007/s12010-022-04134-w 36114924

[B231] Youdim K. A. Martin A. Joseph J. A. (2000). Essential fatty acids and the brain: Possible health implications. Int. J. Dev. Neurosci. 18, 383–399. 10.1016/s0736-5748(00)00013-7 10817922

[B232] Yu Z. Hou Q. Liu M. Xie Z. Ma M. Chen H. (2023). From lab to application: Cultivating limnetic microalgae in seawater coupled with wastewater for biodiesel production on a pilot scale. Water Res. 229, 119471. 10.1016/j.watres.2022.119471 36535089

[B233] Zainuddin N. J. Babji A. S. Said M. (2011). Extraction of lipids and purification of linoleic acid from Clarias macrocephalus oil. Aquac. Aquarium, Conservation Legislation 4, 423–429.

[B234] Zarli A. (2020). “Chapter 6 - oleochemicals: All time players of green chemistry,” in Studies in surface science and catalysis. Editors Basile A. Centi G. Falco M. D. Iaquaniello G. (Amsterdam, Netherlands: Elsevier), 77–95. 10.1016/B978-0-444-64337-7.00006-9

[B235] Zhang G. Liu J. Liu Y. (2013). Concentration of omega-3 polyunsaturated fatty acids from oil of Schizochytrium limacinum by molecular distillation: Optimization of technological conditions. Industrial Eng. Chem. Res. 52, 3918–3925. 10.1021/ie3020044

[B236] Zhang H. Fang T. Pan W. Liao A. Wei Z. (2011). Optimization of the enrichment of alpha-linolenic acid in silkworm pupal oil by the method of urea adduction fractionation. Food Sci. 32, 74–77.

[B237] Zhang H. Gong T. Li J. Pan B. Hu Q. Duan M. (2022). Study on the effect of spray drying process on the quality of microalgal biomass: A comprehensive biocomposition analysis of spray-dried *S. acuminatus* biomass. BioEnergy Res. 15, 320–333. 10.1007/s12155-021-10343-8

[B238] Zhang X. Chen H. Wang H. Wang Q. (2021). Time-course effects of Tris(1,3-dichloro-2-propyl) phosphate (TDCPP) on *Chlorella pyrenoidosa*: Growth inhibition and adaptability mechanisms. J. Hazard. Mater. 402, 123784. 10.1016/j.jhazmat.2020.123784 33254794

[B239] Zhao J. Ma M. Yan X. Zhang G. Xia J. Zeng G. (2022). Expression and characterization of a novel lipase from *Bacillus licheniformis* NCU CS-5 for application in enhancing fatty acids flavor release for low-fat cheeses. Food Chem. 368, 130868. 10.1016/j.foodchem.2021.130868 34438173

[B240] Zheng H. Yin J. Gao Z. Huang H. Ji X. Dou C. (2011). Disruption of *Chlorella vulgaris* cells for the release of biodiesel-producing lipids: A comparison of grinding, ultrasonication, bead milling, enzymatic lysis, and microwaves. Appl. Biochem. Biotechnol. 164, 1215–1224. 10.1007/s12010-011-9207-1 21347653

[B241] Zheng S. Chen S. Zou S. Yan Y. Gao G. He M. (2021). Bioremediation of Pyropia-processing wastewater coupled with lipid production using *Chlorella* sp. Bioresour. Technol. 321, 124428. 10.1016/j.biortech.2020.124428 33272824

[B242] Zheng S. Zou S. Feng T. Sun S. Guo X. He M. (2022). Low temperature combined with high inoculum density improves alpha-linolenic acid production and biochemical characteristics of *Chlamydomonas reinhardtii* . Bioresour. Technol. 348, 126746. 10.1016/j.biortech.2022.126746 35065224

[B243] Zheng Y. Xue C. Chen H. Jia A. Zhao L. Zhang J. (2023). Reconstitution and expression of *mcy* gene cluster in the model cyanobacterium *Synechococcus* 7942 reveals a role of MC-LR in cell division. New Phytol. 2023, 18766. 10.1111/nph.18766 36683448

[B244] Zheng Z. Dai Z. Shen Q. (2018). Enrichment of polyunsaturated fatty acids from seal oil through urea adduction and the fatty acids change rules during the process. J. Food Process. Preserv. 42, e13593. 10.1111/jfpp.13593

[B245] Zhou Y. Chen Y. Li M. Hu C. (2020). Production of high-quality biofuel via ethanol liquefaction of pretreated natural microalgae. Renew. Energy 147, 293–301. 10.1016/j.renene.2019.08.136

[B246] Zhu J. Chen W. Chen H. Zhang X. He C. Rong J. (2016). Improved productivity of neutral lipids in *Chlorella* sp. A2 by minimal nitrogen supply. Front. Microbiol. 7, 557. 10.3389/fmicb.2016.00557 27148237 PMC4838625

[B247] Zhuang Y. Guo J. Chen L. Li D. Liu J. Ye N. (2012). Microwave-assisted direct liquefaction of Ulva prolifera for bio-oil production by acid catalysis. Bioresour. Technol. 116, 133–139. 10.1016/j.biortech.2012.04.036 22609667

[B248] Zou X. Ye L. He X. Wu S. Zhang H. Jin Q. (2020). Preparation of DHA-rich medium- and long-chain triacylglycerols by lipase-catalyzed acidolysis of microbial oil from *Schizochytrium* sp.with medium-chain fatty acids. Appl. Biochem. Biotechnol. 191, 1294–1314. 10.1007/s12010-020-03261-6 32096059

